# Sex- and Age-Specific Prevalence of Osteopenia and Osteoporosis: Sampling Survey

**DOI:** 10.2196/48947

**Published:** 2024-04-05

**Authors:** Yao Fan, Qun Li, Yu Liu, Jing Miao, Ting Zhao, Jinxin Cai, Min Liu, Jun Cao, Haifeng Xu, Lai Wei, Mengxia Li, Chong Shen

**Affiliations:** 1 Division of Clinical Epidemiology Affiliated Geriatric Hospital of Nanjing Medical University Nanjing China; 2 Department of Nutrition The First Affiliated Hospital of Nanjing Medical University Nanjing China; 3 Institute for the Prevention and Control of Chronic Non-communicable Diseases Center for Disease Control and Prevention of Jurong City Jurong China; 4 Department of Clinical Nutrition Affiliated Sir Run Run Hospital of Nanjing Medical University Nanjing China; 5 Department of Epidemiology School of Public Health, Nanjing Medical University Nanjing China

**Keywords:** cross-sectional study, osteopenia, osteoporosis, prevalence, quantitative ultrasound

## Abstract

**Background:**

Osteopenia and osteoporosis are posing a long-term influence on the aging population’s health contributing to a higher risk of mortality, loss of autonomy, hospitalization, and huge health system costs and social burden. Therefore, more pertinent data are needed to demonstrate the current state of osteoporosis.

**Objective:**

This sampling survey seeks to assess the trends in the prevalence of osteopenia and osteoporosis in a Chinese Han population.

**Methods:**

A community-based cross-sectional study involving 16,377 participants used a multistage sampling method. Bone mineral density was measured using the quantitative ultrasonic densitometry. Student *t* test and Mann-Whitney *U* test were used to test the difference between normally and nonnormally distributed quantitative variables between male and female participants. A chi-square (χ^2^) test was used to compare categorized variables. Stratified analysis was conducted to describe the prevalence rates of osteoporosis (T score ≤–2.5) and osteopenia (T score –2.5 to –1.0) across age, sex, calcium intake, and menopause. A direct standardization method was used to calculate the age-standardized prevalence rates of osteoporosis and osteopenia. T-score was further categorized into quartiles (T1-T4) by age- and sex-specified groups.

**Results:**

The prevalence rates of osteopenia and osteoporosis were 40.5% (6633/16,377) and 7.93% (1299/16,377), respectively, and the age-standardized prevalence rates were 27.32% (287,877,129.4/1,053,861,940) and 3.51% (36,974,582.3/1,053,861,940), respectively. There was an increase in osteopenia and osteoporosis prevalence from 21.47% (120/559) to 56.23% (754/1341) and 0.89% (5/559) to 17.23% (231/1341), respectively, as age increased from 18 years to 75 years old. The prevalence rates of osteopenia and osteoporosis were significantly higher in female participants (4238/9645, 43.94% and 1130/9645, 11.72%) than in male participants (2395/6732, 35.58% and 169/6732, 2.51%; *P*<.001), and in postmenopausal female participants (3638/7493, 48.55% and 1053/7493, 14.05%) than in premenopausal female participants (538/2026, 26.55% and 53/2026, 2.62%; *P*<.001)*.* In addition, female participants with a history of calcium intake had a lower osteoporosis prevalence rate than female participants without any history of calcium intake in all age groups (*P*=.004). From low quartile to high quartile of T-score, the prevalence of diabetes mellitus (752/4037, 18.63%; 779/4029, 19.33%; 769/3894, 19.75%; and 869/3879, 22.4%) and dyslipidemia (2228/4036, 55.2%; 2304/4027, 57.21%; 2306/3891, 59.26%; and 2379/3878, 61.35%) were linearly increased (*P*<.001), while the prevalence of cancer (112/4037, 2.77%; 110/4029, 2.73%; 103/3894, 2.65%; and 77/3879, 1.99%) was decreased (*P*=.03).

**Conclusions:**

Our data imply that as people age, osteopenia and osteoporosis are more common in females than in males, particularly in postmenopausal females than in premenopausal females, and bone mineral density significantly affects the prevalence of chronic diseases. These findings offer information that can be applied to intervention programs meant to prevent or lessen the burden of osteoporosis in China.

## Introduction

Osteoporosis is a degenerative condition that affects the entire body’s bones. It is characterized by decreased bone mineral density (BMD) with low bone mass and deterioration of bone tissue microstructure, which makes bones more fragile and increases the risk of fracture [1]. Osteopenia refers to BMD levels that are below the normal range but not as low as osteoporosis [2]. The prevalence rates of osteopenia and osteoporosis are estimated to be 40.40% and 19.75% globally [3], respectively, and their burden has been increasing for nearly 3 decades. From 1990 to 2019, the disability-adjusted life years (DALYs) and global death of both osteopenia and osteoporosis increased by 93.82% and 111.16% globally [4], respectively. The DALY and death count of both osteopenia and osteoporosis-related fractures increased by 121.07% and 148.65%, respectively during the same period, and China is among the top 5 countries with the highest DALYs number in the osteopenia and osteoporosis-related fractures [4].

The risk factors of osteoporosis include family history, abnormal BMI, unhealthy lifestyles, being a woman, and aging [5]. It is estimated that by 2050, the proportion of the older adult population in China will represent 26.1% of the total Chinese population [6]. With the rapid increase in the proportion of older adults globally and in China, osteoporosis might pose a major threat to public health. Osteoporosis frequently induces fragile fractures of the spine, hip, distal forearm, and proximal humerus even with little strength stress force [7,8]. Generally, the clinical symptoms of osteoporosis appear after fracture occurrence and can result in significant disability and excruciating pain, which interferes with normal activities and frequently lowers the quality of life [9,10]. In addition, osteoporosis and its related fractures also increase the risk of mortality, loss of autonomy, nursing home referral, and hospitalization, leading to long-term disability, public health system costs, and a huge social burden [10,11].

It is estimated that by 2050, the annual osteoporosis-related fractures will increase to 5.99 million in China, with a projected cost of US $25.43 billion, representing a 2.7-fold increase compared to 2010 [12]. With the varying socioeconomic conditions, varied lifestyles, and expanding older population in a sizable developing nation like China, more pertinent data are needed to demonstrate the current state of osteoporosis. In addition, the prevalence of aging-related diseases such as type 2 diabetes mellitus (T2DM), coronary heart disease (CHD), and cardiovascular and cerebrovascular diseases (CCVD) is also increasing. These diseases not only often coexist with osteopenia and osteoporosis in older people but also share some common risk factors such as abnormal BMI, unhealthy lifestyles, and aging [5,13,14]. However, the relationship between osteoporosis and T2DM [15,16], CHD [17,18], and CCVD [19,20] remains controversial across studies. Therefore, to aid in the development of health care strategies for the expanding population, high-quality epidemiological studies are required to periodically estimate the prevalence of osteoporosis. Herein, we aimed to investigate the trends in the prevalence of osteoporosis and osteopenia and evaluate the impact of decreased BMD on common chronic diseases in a 2-stage sampling epidemiological survey.

## Methods

### Study Design and Participants

**Figure 1:**
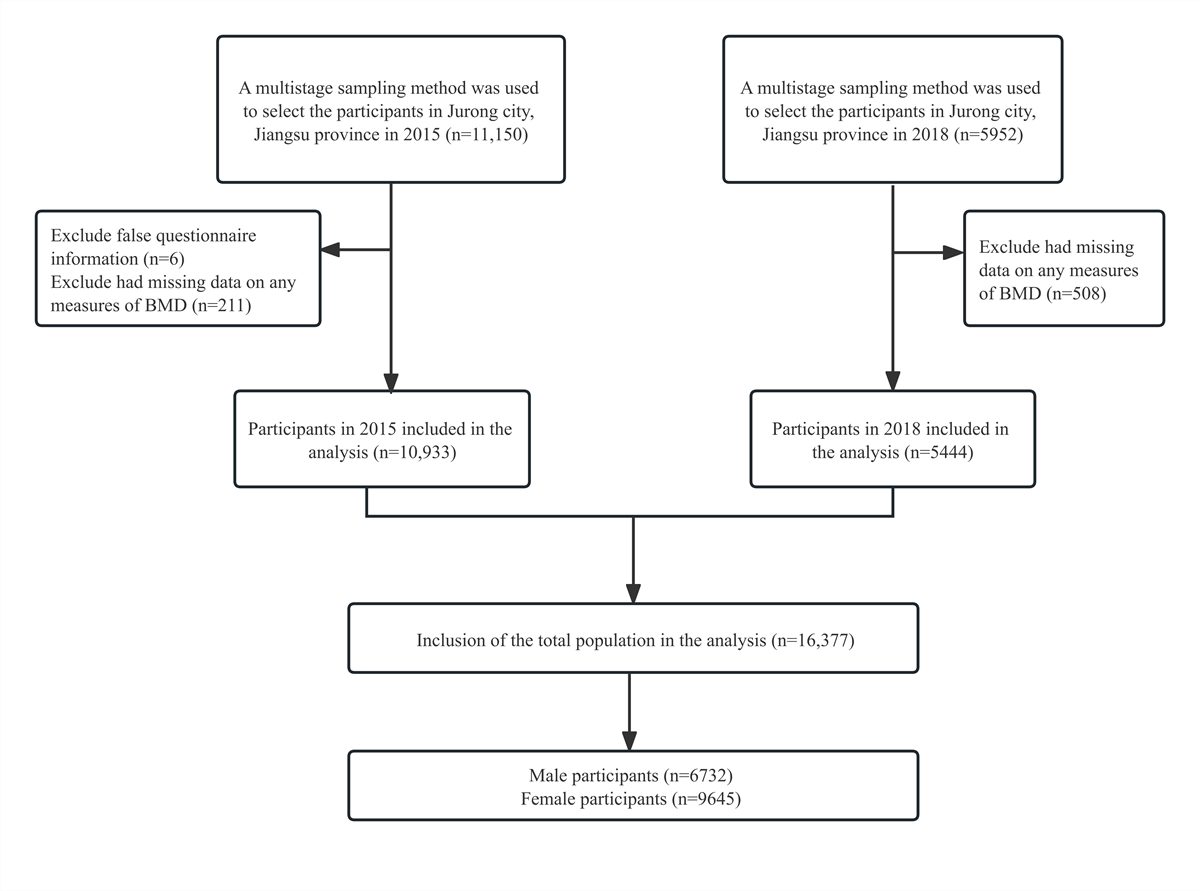
Flowchart of the inclusion of research objects. BMD: bone mineral density.

The baseline survey of the Jurong cohort study was carried out using a multistage sampling method in 16 townships of Jurong City, Jiangsu Province, in the south of China [21]. Eligible participants included were adult inhabitants (who lived locally for more than 6 months in the past 12 months and aged older than 18 years), with over 80% (13,682/17,102) coming from rural areas and having resided locally for more than 6 months in the past year. Individuals not included in this study were those having acute or severe medical conditions and those who were unwilling to take part in the study survey. The recruitment of the study population was done in 2 stages: the first stage included 11,150 participants from October to November 2015 and the second stage included 5952 participants from November to December 2018. Because this study aimed to observe the prevalence of chronic diseases including osteoporosis, stroke, and CHD, the 2-stage sampling was undertaken to have an efficient sample size for the need to estimate risk factors. The participants with missing information (n=6) and without measures of BMD (n=719) were excluded, and finally, 16,377 participants were included in the study (Figure 1).

### Questionnaire

The questionnaire included demographic information, smoking and drinking habits, nutritional status, physical activity (PA), menopausal status, and history of chronic diseases (Multimedia Appendix 1). PA encompasses dynamic behavior and static behavior. Dynamic behaviors include PAs at work, transportation, and leisure time, while static behaviors include total static behavior and spare time static behavior. Using the International Physical Activity Questionnaire as a reference, the level of PA was quantified by metabolic equivalent (MET). The average daily activity duration was calculated by dividing the time of participating in dynamic behavior by 7 times the daily activity duration. Physical activity index (PAI), the ratio of an individual’s total energy consumption and basic metabolic energy consumption in one day, was calculated as follows:



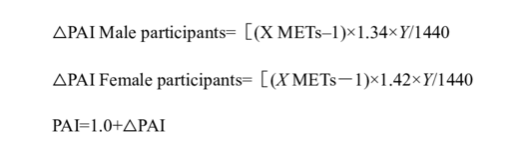



where X is the intensity of an activity and Y is the time for this activity (minutes).

BMI was calculated as the ratio of the weight (kg) to squared height (m^2^). Participants with a BMI of 18.5-24, 24-28, and >28 kg/m^2^ were defined as normal weight, overweight, and obese, respectively [22]. The history of calcium and drug intake and the history of fracture or fall were self-reported by participants. To ensure the accuracy of medication information, participants who were taking drugs were required to bring the package of their medicines.

Menopause is defined as the absence of menstruation for 12 months after the last menstruation or self-reported removal of the uterus or ovaries surgically. The history of CHD, stroke, and cancer were also self-reported by participants. CCVD was defined as a history of CHD, stroke, or both. Hypertension was defined as an average systolic blood pressure/diastolic blood pressure level ≥140/90 mm Hg or self-reported by participants or those taking antihypertensive medicine. Diabetes mellitus (DM) was defined as an average fasting blood glucose ≥7.0 mmol/L, or as self-reported by participants, or those taking hypoglycemic agents or insulin injections. Dyslipidemia was defined as total cholesterol (TC) ≥5.2 mmol/L or low-density lipoprotein cholesterol ≥3.4 mmol/L or high-density lipoprotein cholesterol <1.0 mmol/L or triglyceride (TG) ≥1.7 mmol/L or self-reported participants about the history of dyslipidemia according to the Guidelines for the Prevention and Treatment of Dyslipidemia in Chinese Adults (Revised edition, 2016).

### BMD Measurement

BMD measurement was performed using CM-200. The BMD values of the left side of the heel were measured while seated. Before measurement, the age and sex of participants were entered into the quantitative ultrasound system (QUS), and the instrument automatically converted them to a T-score. The BMD was grouped into 3 based on the updated World Health Organization–recommended criteria: T-score≤–2.5 was defined as osteoporosis, –2.5 to –1.0 was defined as osteopenia, and T-score>–1.0 was defined as normal BMD [23].

### Biochemistry Measurements

After fasting over 8 hours, venous blood was drawn to measure the serum level of high-density lipoprotein cholesterol, TC, TG, and the plasma level of glucose. Low-density lipoprotein (μmol/L) = TC – (high-density lipoprotein + TG/2.2). Blood samples were tested within 4 hours using a Cobas 6000 analyzer (Roche Diagnostics GmbH).

### Statistical Analysis

The normally distributed continuous variables were described by mean (SD). The nonnormally distributed variables were described by the median and IQR. Student *t* test and Mann-Whitney *U* test were used to test the difference of variables between male and female participants. A chi-square (χ^2^) test was used to compare categorized variables. Stratified analysis was conducted to describe the prevalence rates of osteoporosis and osteopenia across age, sex, and calcium intake. Based on the 2010 national census data for the Chinese population, we used a direct standardization method to calculate the age-standardized prevalence rate of osteoporosis and osteopenia. Logistic regression analyses were performed, and the association was estimated with an odds ratio (OR) and 95% confidence interval (CI). T-score was categorized into quartiles (T1-T4) by age- and sex-specified groups. A 2-tailed *P* value less than .05 was considered to indicate statistical significance. All statistical analyses were performed using SPSS Statistics (version 24.0; IBM Corp).

### Ethical Considerations

Nanjing Medical University’s Research Ethics Committee approved the study (2015077). The study was performed following the Declaration of Helsinki. The study’s scope, objectives, and advantages were thoroughly communicated to people who were invited to participate. All survey participants gave written informed consent. The collected information was kept confidential and used solely for research purposes.

## Results

### General Characteristics of Participants

Of the 16,377 participants included in this study, 6732 (41.1%) were male participants with an average age of 61.51 (10.84) years, and 9645 (58.9%) were female participants with an average age of 59.48 (SD 10.93) years. Participants with chronic diseases were identified, including 1287 (7.9%) with fracture or fall, 9558 (58.4%) with hypertension, 3113 (19%) with DM, 9523 (58.2%) with dyslipidemia, 267 (1.6%) with CHD, 890 (5.4%) with strokes, and 439 (2.7%) with cancer. In total, 8709 (53.4%) individuals self-reported a history of taking drugs in the past year, and among those, 766 (4.7%) people had taken calcium tablets. About 77.7% (7493/9645) of the female participants were postmenopausal, with 3.3% (247/7493) having their uterus or ovaries removed surgically (Table 1).

**Table 1:** Characteristics of the study participants.

Variables		Total population (N=16,377)	Male participants (n=6732)	Female participants (n=9645)	*P* value	
Age (years), mean (SD)		60.32 (10.94)	61.51 (10.84)	59.48 (10.93)	<.001	
Education, n (%)						<.001
	Illiteracy or primary	9819 (60)	3148 (46.8)	6671 (69.3)		
	Junior high school	4838 (29.6)	2514 (37.4)	2324 (24.1)		
	Senior high school or above	1699 (10.4)	1062 (15.8)	637 (6.6)		
Marital status, n (%)						<.001
	Unmarried	246 (1.5)	205 (3)	41 (0.4)		
	Married	14,134 (86.3)	5972 (88.7)	8162 (84.6)		
	Divorced or widowed	1997 (12.2)	555 (8.2)	1442 (15)		
Occupation, n (%)						<.001
	Manual worker	11,983 (73.2)	5470 (83.3)	6513 (68.1)		
	Technician	323 (2)	202 (3.1)	121 (1.3)		
	Businessman	551 (3.4)	346 (5.3)	205 (2.1)		
	Other	3272 (20)	550 (8.4)	2722 (28.5)		
Income (CNY^a^), median (IQR)		2500 (1250-5000)	2500 (1250-4583)	2917 (1250-5000)	.13	
Smoking, n (%)		3651 (22.3)	3522 (52.3)	129 (1.3)	<.001	
Drinking, n (%)		4542 (27.7)	3673 (54.6)	869 (9)	<.001	
Physical activity index, median (IQR)		4 (1.28-16.00)	3.36 (1.18-16.00)	5.03 (1.37-14.67)	<.001	
History of fracture or fall, n (%)		1287 (7.9)	504 (7.5)	783 (8.1)	.14	
BMI (kg/m^2^), mean (SD)		24.99 (3.38)	24.74 (3.26)	25.17 (3.44)	<.001	
History of taking calcium, n (%)		766 (4.7)	197 (2.9)	569 (5.9)	<.001	
History of taking drugs, n (%)		8709 (53.4)	3605 (53.7)	5104 (53.4)	.61	
Postmenopausal in female participants, n (%)		N/A^b^	N/A	7493 (77.7)	N/A	
FBG^c^ (mmol/L), mean (SD)		6.21 (1.86)	6.23 (1.89)	6.20 (1.84)	.36	
TC^d^ (mmol/L), mean (SD)		5.06 (0.93)	4.95 (0.91)	5.13 (0.93)	<.001	
TG^e^ (mmol/L), mean (SD)		1.66 (1.29)	1.63 (1.42)	1.68 (1.19)	.02	
HDL-C^f^ (mmol/L), mean (SD)		1.52 (0.42)	1.52 (0.47)	1.52 (0.38)	.67	
LDL-C^g^ (mmol/L), mean (SD)		2.83 (0.79)	2.74 (0.78)	2.88 (0.80)	<.001	
HTN^h^, n (%)		9558 (58.4)	4220 (62.7)	5338 (55.3)	<.001	
T2DM^i^, n (%)		3113 (19)	1259 (18.7)	1854 (19.2)	.14	
Dyslipidemia, n (%)		9523 (58.2)	3651 (54.2)	5872 (60.9)	<.001	
CHD^j^, n (%)		267 (1.6)	133 (2)	134 (1.4)	.004	
Stroke, n (%)		890 (5.4)	389 (5.8)	501 (5.2)	.11	
Cancer, n (%)		439 (2.7)	227 (3.4)	212 (2.2)	<.001	

^a^A currency exchange rate of 1 CNY= US $0.14 is applicable.

^b^N/A: not applicable.

^c^FBG: fasting blood glucose.

^d^TC: total cholesterol.

^e^TG: triglycerides.

^f^HDL-C: high-density lipoprotein cholesterol.

^g^LDL-C: low-density lipoprotein cholesterol.

^h^HTN: hypertension.

^i^T2DM: type 2 diabetes mellitus.

^j^CHD: coronary heart disease.

Male participants had higher prevalence of hypertension (4220/6732, 62.7%), CHD (133/6732, 2%), and cancer (227/6732, 3.4%) than female participants (5338/9645, 55.3%; 134/9645, 1.4%; and 212/9645, 2.2%) at a significant level of *P*<.05 but had a lower prevalence of dyslipidemia (3651/6730, 54.2%) than female participants (5872/9640, 60.9%; *P*<.05). As expected, male participants had a higher proportion of smoking (3522/6732, 52.3%) and drinking (3673/6732, 54.6%) than female participants (129/9644, 1.3%; and 869/9643, 9%; *P*<.001). Male participants had lower levels of PAI (median 3.36, IQR 1.18-16.00), BMI (mean 24.74, SD 3.26 kg/m^2^), TC (mean 4.95, SD 0.91 mmol/L), and TG (mean 1.63, SD 1.42 mmol/L) than female participants (median 5.03, IQR 1.37-14.67; mean 25.17, SD 3.44 kg/m^2^; mean 5.13, SD 0.93 mmol/L; and mean 1.68, SD 1.19 mmol/L; *P*<.05).

### Prevalence of Osteopenia and Osteoporosis Among Age Groups

**Figure 2:**
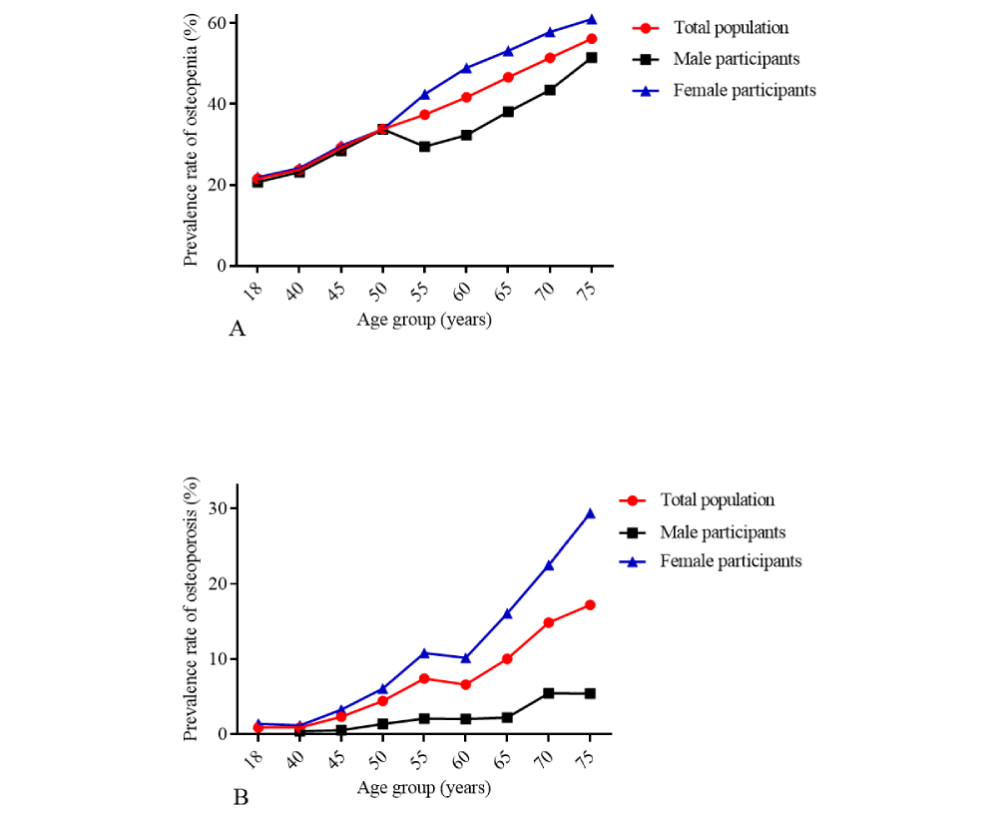
Sex-specific prevalence changes of osteopenia and osteoporosis with age. In this cross-sectional study, the age-specific prevalence of osteoporosis and osteopenia were calculated for the 16,377 Chinese adults who constitute the overall study population and further separately for 6732 male participants and 9645 female participants. The prevalence rate of osteopenia significantly increased with the age in male participants, female participants, and the whole study population (A), all Ptrend values were less than .001, and the results were statistically significant. The prevalence rates of osteopenia showed a significant statistical difference between male and female participants in the age group 55 years, 60 years, 65 years, 70 years, and 75 years, and all *P* values were less than .001. The prevalence rate of osteoporosis significantly increased with the age in male participants, female participants, and the whole study population (B), all Ptrend values were less than .001, and the results were statistically significant. The prevalence rates of osteoporosis showed a significant statistical difference between male and female participants in the 45 years, 50 years, 55 years, 60 years, 65 years, 70 years, and 75 years age groups; all *P* values were less than .001.

The prevalence rates of osteopenia and osteoporosis were 40.5% (6633/16,377) and 7.93% (1299/16,377), respectively. The age-standardized prevalence rates of osteopenia and osteoporosis were 27.32% (287,877,129.4/1,053,861,940) and 3.51% (36,974,582.3/1,053,861,940), respectively. In the population aged 50 years and older, the age-standardized prevalence rates of osteopenia and osteoporosis were 42.34% (142,938,163.8/337,624,151) and 8.96% (30,253,250.83/337,624,151), respectively. There was an increase in osteopenia prevalence rate from 21.47% (120/559) in the 18-year-old group to 56.23% (754/1341) in the 75-year-old group (Figure 2A)*.* Meanwhile, the prevalence rate of osteoporosis increased from 0.89% (5/559) in the 18-year-old group to 17.23% (231/1341) in the 75-year-old group (Figure 2B).

### Sex-Specific Prevalence Changes of Osteopenia and Osteoporosis With Age

The prevalence rates of osteopenia and osteoporosis among female participants (4238/9645, 43.94% and 1130/9645, 11.72%) were higher than in male participants (2395/6732, 35.58% and 169/6732, 2.51%, respectively; *P*<.001). In the study population, the age-standardized prevalence rates of osteopenia and osteoporosis were 22.38% (119,148,921.4/532,444,837) and 0.94% (5,022,330.82/532,444,837) for male participants, while it was 30.97% (161,470,215.4/521,417,103) and 5.52% (28,781,912.82/521,417,103) for female participants. In the population aged 50 years and older, the age-standardized prevalence of osteopenia and osteoporosis was 36.1% (60,821,276.55/168,477,281) and 2.65% (4,471,495.817/168,477,281) for male participants and 47.14% (79,727,904.8/169,146,870) and 14.26% (24,113,222.94/169,146,870) for female participants. Among male participants, the observed prevalence rates of osteopenia (Figure 2A) and osteoporosis (Figure 2B) were 20.71% (41/198) and 0% (0/198) in the 18-year-old group and increased to 51.54% (351/681) and 5.43% (37/681) in the 75-year-old group, respectively. Among female participants, the observed prevalence rates of osteopenia (Figure 2A) and osteoporosis (Figure 2B) were 21.88% (79/361) and 1.39% (5/361) in the 18-year-old group and increased to 61.06% (403/660) and 29.39% (194/660) in the 75-year-old group, respectively. The prevalence rates of osteopenia and osteoporosis among age groups are shown in Multimedia Appendix 2.

### Prevalence of Osteopenia and Osteoporosis Between Postmenopausal and Premenopausal Female Participants

**Figure 3:**
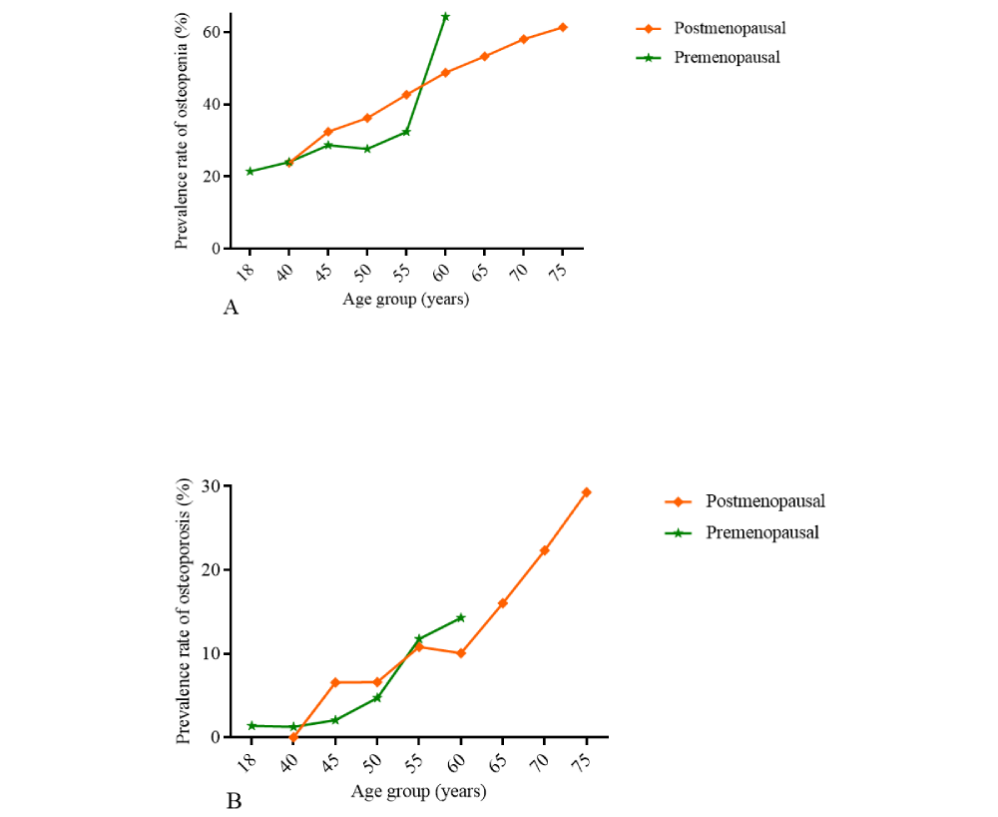
Prevalence of osteopenia and osteoporosis between postmenopausal and premenopausal female participants. In this cross-sectional study, the age-specific prevalence of osteoporosis and osteopenia was calculated for 7493 Chinese postmenopausal female participants and 2162 premenopausal female participants. The prevalence rates of osteopenia significantly increased with age in premenopausal and postmenopausal female participants, and all Ptrend values were less than .001, the results were statistically significant (A). Prevalence of osteopenia showed a significant statistical difference between premenopausal and postmenopausal female participants in the age group 50 years, *P* value was less than .001. The prevalence rate of osteoporosis significantly increased with age in premenopausal and postmenopausal female participants and all Ptrend values were less than .001, the results were statistically significant (B). Prevalence rates of osteoporosis showed a significant statistical difference between premenopausal and postmenopausal female participants in the age group 50 years (*P*<.05).

The prevalence rates of osteopenia and osteoporosis in postmenopausal female participants were higher (3638/7493, 48.55% and 1053/7493, 14.05%) than in premenopausal female participants (538/2026, 26.55% and 53/2026, 2.62%; *P*<.001). In premenopausal female participants, the observed prevalence of osteopenia was 21.41% (76/355) among the 18-year-old group and increased as the age increased. In postmenopausal female participants, the observed prevalence of osteopenia was 23.81% (5/21) in the 40-year-old group and increased to 61.37% (394/642) in the 75-year-old group (Figure 3A).

In premenopausal female participants, the observed prevalence of osteoporosis was 1.25% (5/400) among the 40-year-old group and increased to 14.29% (2/14) among the 60-year-old group. In postmenopausal female participants, the observed prevalence of osteoporosis was 6.56% (17/259) among the 45-year-old group and increased to 29.28% (188/642) in the 75-year-old group (Figure 3B). The prevalence rates of osteopenia and osteoporosis in postmenopausal and premenopausal female participants are shown in Multimedia Appendix 3.

### Prevalence of Osteopenia and Osteoporosis in the Subgroups With and Without a History of Calcium Intake

**Figure 4:**
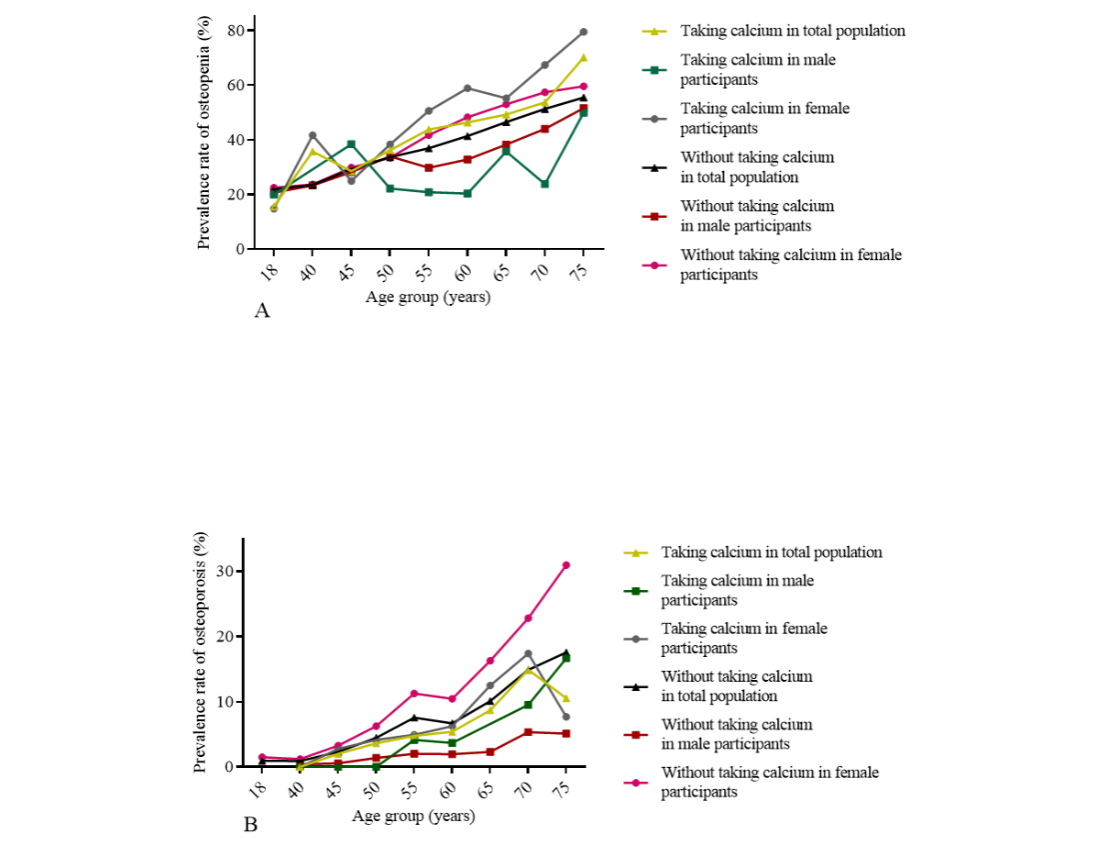
Prevalence of osteopenia and osteoporosis in the subgroups with and without a history of calcium intake. In this cross-sectional study, the age-specific prevalence of osteoporosis and osteopenia were calculated for 766 Chinese adults taking calcium and separately for 197 male participants and 569 female participants as well as for 15,581 Chinese adults not taking calcium and separately for 6527 male participants and 9054 female participants. The prevalence rate of osteopenia significantly increased with the age in the whole study population, male, and female participants with and without a history of taking calcium (A). All Ptrend values were less than .001, the results were statistically significant except in male participants with a history of taking calcium. The prevalence rates of osteoporosis significantly increased with the age in the whole study population, male and female participants with and without a history of taking calcium (B). All Ptrend values were less than .001, the results were statistically significant. The prevalence rates of osteopenia and osteoporosis showed no significant statistical difference between participants with and without a history of taking calcium in all age groups.

Among participants with and without a history of calcium intake, the prevalence rate of osteopenia and osteoporosis was 44.52% (341/766), 40.28% (6276/15,581), 6.27% (48/766), and 8.02% (1250/15,581), respectively. For male participants, the prevalence rate of osteopenia was lower among those taking calcium (55/197, 27.92%) than those not taking calcium (2339/6527 35.84%; *P*=.03). The prevalence rate of osteopenia was higher among female participants taking calcium (286/569, 50.26%) than those not taking calcium (3937/9054, 43.48%; *P*=.04; Figure 4A). The prevalence rates of osteoporosis among the overall study participants taking calcium and not taking calcium were 4.06% (8/197) and 2.47% (161/6527) in male participants and 7.03% (40/569) and 12.03% (1089/9054) in female participants, respectively, with significant differences among female participants (*P=*.004; Figure 4B). The prevalence rates of osteopenia and osteoporosis in subgroups with and without taking calcium are shown in Multimedia Appendix 4.

### Association Analysis of Taking Calcium With Osteopenia and Osteoporosis

After adjustment for age, sex, PAI, occupation, history of hypertension, history of T2DM, history of dyslipidemia, history of CHD, history of stroke, and history of cancer, there was no significant association between calcium intake and osteopenia as well as osteoporosis, and the corresponding ORs (95% CIs) were 1.10 (0.94-1.29) and 0.90 (0.61-1.34), respectively.

Furthermore, sex-stratified analysis showed that calcium intake increased the risk of osteopenia in female participants (OR 1.26, 95% CI 1.04-1.52; Multimedia Appendix 5) after adjusting age, PAI, occupation, history of hypertension, history of T2DM, history of dyslipidemia, history of CHD, history of stroke, and history of cancer.

### Prevalence of Chronic Disease Among Different T-Score Groups

In the total study population, the prevalence of DM, dyslipidemia, CHD, and CCVD differed among the low, medium-low, medium-high, and high quartiles of the T-score. From the low quartile to the high quartile of the T-score, the prevalence rates of DM (752/4037, 18.63%; 779/4029, 19.33%; 769/3894, 19.75%; and 869/3879, 22.4%) and dyslipidemia (2228/4036, 55.2%; 2304/4027, 57.21%; 2306/3891, 59.26%; and 2379/3878, 61.35%) were linearly increased (*P*<.001), while the prevalence rate of cancer (112/4037, 2.77%; 110/4029, 2.73%; 103/3894, 2.65%; and 77/3879, 1.99%) was decreased (*P*=.03). The prevalence rate of CHD was 1.29% (52/4037), 1.54% (62/4029), 2.16% (84/3894), and 1.99% (77/3879) from low quartile to high quartile. The prevalence rate of CCVD was 6.47% (261/4037), 6.3% (254/4029), 6.91% (269/3894), and 7.76% (301/3879) from the lowest quartile to the highest quartile (Multimedia Appendix 6).

Further stratified analysis by BMI showed that in the normal weight group, the prevalence rates of DM were 13.49% (241/1786), 13.03% (214/1642), 13.27% (206/1552), and 16.25% (217/1335) among the lowest, second, third, and highest quartile values of the T-score. In the overweight group, the prevalence rate of dyslipidemia increased from 58.99% (968/1641) to 64.63% (1120/1733), with values of T-score increasing. In the obesity group, the prevalence rates of dyslipidemia were 64.75% (395/610), 71.33% (510/715), 72.83% (520/714), and 71.6% (580/810), and the prevalence rates of CHD were 0.82% (5/610), 2.09% (15/716), 2.66% (19/714), and 3.46% (28/810) from the lowest quartile to the highest quartile. All the *P* values were less than .05 (Multimedia Appendix 7).

## Discussion

### Principal Findings

With a growing older adult population, the public health issue caused by osteoporosis and osteopenia is worthy of attention. However, the prevalence of osteoporosis varied across different studies because of inconsistent measuring instruments [24], bone sites [25], age group, sample size, region [26], and diagnosis criteria. This study investigated the prevalence of osteoporosis and osteopenia in a relatively large sample size Chinese population and explored the impact of chronic common disorders on BMD. With these results, interventions can be carried out for the target population to reduce the burden that osteoporosis poses to public health.

In this study, the age-standardized prevalence rates of osteoporosis and osteopenia were 3.51% (36,974,582.3/1,053,861,940) and 27.32% (287,877,129.4/1,053,861,940), respectively. Similarly, Qiao et al [27] analyzed the prevalence of osteoporosis and osteopenia using the same measuring instruments at the same bone site with this study in rural areas of Henan province in Middle-Eastern China and reported the age-standardized prevalence rates to be 11.76% and 42.09%, respectively. Another study reported that Shanghai had the lowest prevalence rates of osteoporosis and osteopenia compared with other regions in China [28]. A possible reason for the differences in the prevalence of osteopenia and osteoporosis is that each province in China may have different lifestyle-related characteristics that are population specific due to educational, sociocultural, and geographic considerations. For instance, a study has shown that provinces where alcohol is produced, such as Jiangsu, Anhui, Shandong, and Sichuan, had higher alcohol consumption proportions compared with the national level [29]. Meanwhile, lifestyle factors including alcohol are associated with poor bone health [30].

Similarly, Zeng et al [31] measured the BMD values using GE Lunar dual-energy X-ray absorptiometry (DXA) in China and estimated the age-standardized prevalence rate of osteoporosis in male and female participants older than 50 years old to be 6.46% and 29.13%, respectively. Wang et al [32] measured the BMD values using DXA and reported that the prevalence rate of osteoporosis among male participants aged 40 years or older was 5% and 20.6% among female participants of the same age group in China. A previous study has shown that QUS and X-ray density measurement BMD methods (DXA and central quantitative computed tomography) showed the same ability to distinguish between normal populations and osteoporosis [33]. Many epidemiological studies use QUS as a screening tool for osteoporosis because it is easier to expand the sample size, simplify the measuring method, and reduce the cost [34,35]. Other important prospective studies have assessed fracture risk through calcaneal QUS [36,37]. The EPIC-Norfolk prospective population study [37] demonstrated that measuring BMD by QUS at the calcaneus can effectively predict fracture risk in the future. It is worth noting that the QUS T-score may be more adaptable for clinically detecting the risk of osteoporosis in female patients [38]. However, the age-standardized prevalence of osteoporosis at different sites (lumbar spine, femoral neck, and total femur) is also different [31]. Wang et al [39] and Chen et al [40] reported gaps in the prevalence of osteoporosis. Wang et al [28] detected the BMD in the lumbar vertebra and left hip joint by DXA and also confirmed that the prevalence of osteoporosis would be greatly reduced if diagnosed with BMD in every single region only. It is worth noting that the prevalence rates of postmenopausal female participants in this study are close to the results of Wang et al [28], which diagnosed hip BMD. Thus, we speculated that the relative differences in prevalence rates may be due to different measurement regions and a single region. The prevalence rates of osteopenia and osteoporosis in the current population were moderate compared to the previous studies. Importantly, the age-standardized prevalence rates of osteopenia and osteoporosis were higher (79,727,904.8/169,146,870, 47.14% and 24,113,222.94/169,146,870, 14.26%) in female participants aged 50 years and older.

There are significant sex differences in the prevalence and pathogenesis of osteoporosis [41]. Previous studies found that osteoporosis was more prevalent among female than male participants, especially among postmenopausal female participants [27,31]. For menopausal females, the sudden change in hormonal levels can contribute to increased bone loss. Our results are consistent with the previous reports.

A prospective cohort study in an Italian population assessed the association between dietary calcium intake and osteoporosis and a fragility fracture. Their results indicated that low calcium intake was a risk factor for low BMD in Italy [42]. However, in one of the central populations of the Canadian Multicentre Osteoporosis Study, it was found that older female participants taking calcium supplements had no significant BMD preservation [43]. In this study, the prevalence of osteopenia was higher in female participants taking calcium than in those who did not. That may be attributed to the fact that more patients with osteopenia receive diagnosis and treatment advice and take a calcium supplement. Moreover, considering the design of our study is a cross-sectional study, a causal association between calcium supplementation and BMD cannot be stated.

This study indicated that patients with T2DM, dyslipidemia, CHD, and CCVD were associated with higher BMD. In reality, previous observations reported that the association of BMD value with T2DM was inconclusive [15,16]. Recently, Wang et al [28] also found that T2DM was associated with higher BMD. Even some large clinical studies revealed the same results [44,45]. Some researchers have speculated the anabolic effect of insulin on bone tissue [46]. Hyperinsulinemia caused by insulin resistance in T2DM may negatively affect sex hormone–binding globulin, leading to higher free sex hormone levels that may protect patients with T2DM from bone loss [47,48]. However, animal models have indicated that although bone density is greater in diabetes, the bone structure is more fragile, with fractures occurring under a smaller load and the bones exhibiting reduced mechanical indices [49]. Thus, even though the BMD was high in T2DM, it still should pay more attention to patients with T2DM.

As a marker of bone turnover, osteoprotegerin (OPG) can reflect the level of bone metabolism and is used to treat osteoporosis. The animal study results found that OPG-deficient mice develop osteoporosis and premature arterial calcification, suggesting that the OPG system affects vascular calcification [50]. The human study showed that increased OPG levels were associated with the risk of fatal stroke and raised the possibility that the OPG system may be involved in vascular calcification [51]. However, this study was unable to confirm the association between BMD and stroke, although the results indicated the association between BMD and CHD and CCVD.

The findings of our investigation have significant implications for public health. With the increase of Chinese older adults, the prevalence and burden of osteoporosis and osteopenia are expected to increase in the coming years. This study offers information that can be applied to intervention programs meant to prevent or lessen the burden of osteoporosis in China.

### Strengths and Limitations

This study has a relatively large sample size of a Chinese population involving a fairly broad spectrum of ages taken to boost the statistical power. The study also explored the trend of prevalence of osteopenia and osteoporosis across different age groups and identified high-risk individuals and some protective factors such as early calcium supplementation. However, this study has some limitations. First, a causal association between calcium and osteoporosis cannot be drawn since this was a cross-sectional study. A distinct temporal order in which the cause comes before the result is necessary for causality. It is difficult to discern the direction of the relationship when using cross-sectional research because they can only reveal associations at a single point in time. Although cross-sectional research can yield useful findings, it can only reveal information regarding potential protective or risk factors. Therefore, to support the findings of this investigation, experimental studies should be carried out. Second, the average age of the participants in this study was 60 years, while the number of individuals younger than 40 years old was relatively small. Thus, the estimated prevalence rate of young people may not be sufficient and stable; further studies involving a larger number of participants aged younger than 40 years old might verify the prevalence of osteoporosis and osteopenia for that age group. Third, patients with chronic diseases affecting BMD were self-reported and did not include patients who were bedridden for a long time, which might underestimate the prevalence rates of osteoporosis and osteopenia. Despite this, the necessity of a positive response to promoting bone health and intervention measures for osteopenia and osteoporosis is more strongly suggested.

### Conclusions

Our findings suggest that as age increases, females had a higher increase in the prevalence of osteopenia and osteoporosis than males. Importantly, postmenopausal females had higher prevalence rates of osteopenia and osteoporosis than premenopausal females. Higher BMD is associated with an increased risk of DM, dyslipidemia, CHD, and CCVD but a decreased risk of cancer. This research will help develop aging coping strategies to promote bone health and prevent osteoporosis.

## Appendix

Multimedia Appendix 1 Questionnaire.

Multimedia Appendix 2 Prevalence rates of osteopenia and osteoporosis among age-groups.

Multimedia Appendix 3 Prevalence rates of osteopenia and osteoporosis in postmenopausal and premenopausal female participants.

Multimedia Appendix 4 Prevalence rates of osteopenia and osteoporosis in subgroups with and without taking calcium.

Multimedia Appendix 5 Association analysis of history of taking calcium with osteopenia and osteoporosis.

Multimedia Appendix 6 Prevalence of chronic disease among different T-score levels.

Multimedia Appendix 7 Prevalence of chronic disease among different T-score levels.

## Abbreviations

BMD: bone mineral density
CCVD: cardiovascular and cerebrovascular disease
CHD: coronary heart disease
DALY: disability-adjusted life-year
DXA: dual-energy X-ray absorptiometry
MET: metabolic equivalent
OPG: osteoprotegerin
OR: odds ratio
PA: physical activity
PAI: physical activity index
QUS: quantitative ultrasound system
T2DM: type 2 diabetes mellitus
TC: total cholesterol
TG: triglyceride

## Data Availability

The data sets generated and analyzed during this study are available from the corresponding author upon reasonable request.
