# Supplementary material for: Sex- and Age-Specific Prevalence of Osteopenia and Osteoporosis: Sampling Survey
Source: JMIR Public Health Surveill. 2024 Apr 5;10:e48947. doi: 10.2196/48947 (PMC11031699; doi:10.2196/48947)
Supplement: Multimedia Appendix 1 [file publichealth_v10i1e48947_app1.pdf]

根据中华人民共和国《统计法》第三章第十五条规定，“属于私人、家庭的单项调查资料，非经本人同意，不得外泄”。

句容市社区诊断居民健康调查表

（2015）

家庭问卷

该调查对象为：1 随访个案 2 新增抽样个案	
不干胶编码条粘贴处	
家庭问卷调查对象姓名：________	电话号码（手机）：________
监测点(基层医疗机构)名称：	监测点编码：□□
行政村（居委会）名称：	行政村（居委会）编码：□□
村民组（居民组）名称：	村民组（居民组）编码：□□
是否为置换后家庭户<br>1 是 2 否	家庭编码： □□
**调查员在开始家庭问卷调查前填写以上信息**	
调查员签名：________<br>日期：□□□□ 年 □□ 月 □□ 日	监测点质控员签名：________<br>日期：□□□□ 年 □□ 月 □□ 日
市级质控员签名：________ 日期：□□□□ 年 □□ 月 □□ 日	
**调查员和质控员在完成家庭问卷调查后填写以上 3 部分信息**	

句容市疾病预防控制中心

二〇一五年十月

句容市 2015 年社区诊断居民健康调查

知 情 同 意 书

随着我国经济的快速增长、人民生活水平的提高、饮食结构的改变和人口迅速老龄化,以高血压、糖尿病、恶性肿瘤等为代表的慢性非传染性疾病（以下简称“慢性病”）的流行已经成为严重的公共卫生问题。各类慢性病的高致残率和死亡率，为社会、家庭和个人带来沉重的经济负担。我们邀请您参加本次居民慢性病健康调查，内容包括问卷调查、人体测量和血液检测。

项目目的：筛查主要慢性病危险因素，了解重点慢性病的发病情况，了解影响您健康的环境危险因素和遗传危险因素。也可以为您提供慢病基础信息和变化状况信息。

益处：人体测量及血液检测可及早发现高血压，高脂血症，糖尿病和肥胖等慢性病危险因素，还可以使您掌握自己的健康变化趋势，并可提供给你血压、血糖、血脂等指标的测定结果。

可能风险：抽血时，您会有轻微疼痛。

选择性和权利：您可以选择参与或不参与本研究。如果您参加本调查，医生可以帮你测量身高、体重、腰围、臀围、颈围、血压、心率、体脂含量、体能、肌力和骨密度，检测血常规、空腹血糖（FBG）、甘油三酯（TG）、总胆固醇（TC）、高密度脂蛋白胆固醇（HDL-C）、血尿素氮（BUN）、血尿酸（UA）、肌酐（CREA）和总胆红素（T-BIL），并进行慢性病防治知识的宣传。

隐私保护：我们会保护您的私人信息。有关任何个人隐私信息如姓名、年龄、性别等不会被发表出现在任何刊物上，也不会泄露给其他人。

检查费用：上述调查和体检内容不收取任何费用。

签名：我阅读了该知情同意书，愿意参与本项目。

受访对象（签名）：　　　　代理者（签名）：　　　　日期：2015 年　　月　　日

我已向该同志介绍了研究主要内容并说明了参加研究是完全自愿的。

医务人员（签名）：　　　　　　　　　　　　日期：2015 年　　月　　日

家庭登记表

（随访调查对象不需填写此页）

在以下家庭成员登记表中的每一行填写一位符合常住居民条件的家庭成员的信息。按照先男性，后女性，年龄分别从大到小填写。

参考家庭登记表 HH4 年龄，如果年龄大于或等于 18 岁，在 HH5 填写 1，如果年龄不满 18 岁，在 HH5 填写 2。

将 HH5 填写 1 的家庭成员按先男性，后女性，年龄从大到小排序编号，填写在 HH6 中。

姓名<br>(HH1)	与户主的关系代码<br>(HH2)	性别<br>1=男 2=女<br>(HH3)	年龄<br>(HH4)	年龄是否为 18 岁及以上<br>1=是 2=否<br>(HH5)	家庭成员编号<br>(HH6)

注：与户主的关系代码：1=户主，2=配偶，3=儿子或女儿，4=儿媳或女婿，5=孙子女或外孙子女，6=父母，7=公婆/岳父母，8=兄弟或姐妹，9=祖/外祖父母，10=其他亲属，11=无亲属关系（朋友、服务人员、寄宿者、投宿者、其他）

查询分配给该家庭的 KISH 表，选定接受个人调查表调查的家庭成员。

HH7	分配给该家庭的 KISH 表<br>A、B1、B2、C、D、E1、E2、F	□□
HH8	入选的家庭成员编号	□□

家庭饮食和燃料使用情况

家庭饮食状况			
HH9a	您家通常有几人在家吃早餐?	□□人	
HH9b	您家通常有几人在家吃午餐?	□□人	
HH9c	您家通常有几人在家吃晚餐?	□□人	
**HH9a ~HH9c 中"通常"指：目前最一般的情况，如目前一周内，工作日与周末情况不同，则该填写工作日的情况。**			
HH10	您家里通常一个月食用多少植物油？<br>**调查员注意：若不知道或记不清，在小数点前靠右填"-9"，下同。**	□□. □斤	
HH11	您家里通常一个月食用多少动物油？	□□. □斤	
HH12	您家里通常一个月食用多少食盐?	□□. □斤	
HH13	您家里通常一个月食用多少酱油?	□□. □斤	
HH14	您家里通常一个月食用多少糖？	□□. □斤	
HH15	您家里通常一个月食用多少咸菜?	□□. □斤	
HH16	您家里通常一个月食用多少泡菜?	□□. □斤	
HH17	您家里通常一个月食用多少腐乳？	□□. □斤	
HH18	您家里通常一个月食用多少酱(如黄酱、大酱、豆瓣酱等)？	□□. □斤	
**HH10~HH18 填写规则：若"不知道"在小数点前靠右填 "-9"，小数点后用"0"补足。**			
HH19a	您家里是否收到过控盐勺？	1 是<br>2 否 ……………………..…➔<br>99 不清楚 ……..………..…➔	 HH20a<br>HH20a
**控盐勺：烹饪时控制食盐使用量的工具**			
HH19b	如果是，您是从哪里获取的呢？	1 居委会/村委会发放<br>2 自己购买<br>3 亲友赠送<br>88 其他，请说明____________	
HH20a	您家里是否收到过控油壶？	1 是<br>2 否 ……..…………..…➔<br>99 不清楚 …...……………..…➔	 HH21<br>HH21
**控油壶：烹饪时控制控油壶使用量的工具**			
HH20b	如果是，您是从哪里获取的呢？	1 居委会/村委会发放<br>2 自己购买<br>3 亲友赠送<br>88 其他，请说明____________	

HH21	您家里谁主要负责购买食物(填写家庭成员编号)？<br>**调查员注意：若不知道，填“99”；若负责购买食物者没有家庭成员编号，填“88”。**	□□
	**此处填写家庭问卷 HH6 问题中成员的编码。**	
**燃料使用状况**		
HH22	您家现在做饭用的最主要燃料是？	1 柴草/炭/木头/动物粪便<br>2 煤<br>3 煤气/液化气/天然气/沼气<br>4 太阳能/电<br>88 其他，请说明____________
	**若有两种或以上主要燃料，以使用频率最高的为主。**	
HH23	您家做饭时是否使用抽油烟机或排风扇？	1 是 2 否
HH24	您家做饭的地点是？	1 起居室（客厅或卧室）<br>2 单独的厨房<br>3 室外<br>88 其他：请说明____________
	**起居室：指睡觉、休息的房间，如卧室或客厅**<br>**单独的厨房：独立的一间专门用于做饭的房间**<br>**室外：不在室内的，露天的做饭地点**	

家庭经济状况

HH25	上一年您家的总收入是多少？<br>**调查员注意：年收入和月收入只记录其中 1 项。**	1 □□ 元/月 或<br>2 □□ 元/年<br>99 不知道具体收入额<br>97 拒绝回答
	**农村居民家庭记录扣除生产资料投入后的家庭纯收入。**<br>**若子女不与父母同住，子女给父母的赡养费应该算作家庭收入。**<br>**不知道或不清楚具体收入圈选“99”，拒绝回答圈选“97”。**	
HH26	上一年您家的总支出是多少？<br>**调查员注意：年支出和月支出只记录其中 1 项。**	1 □□ 元/月 或<br>2 □□ 元/年<br>99 不知道具体支出额<br>97 拒绝回答
	**家庭生活消费支出包括食品、衣着、居住、家庭设备和用品及服务、医疗保健、交通通讯、文教娱乐用品及服务、其他商品和服务等的支出总额。**<br>**不知道或不清楚具体收入圈选“99”，拒绝回答圈选“97”。**	

根据中华人民共和国《统计法》第三章第十五条规定，“属于私人、家庭的单项调查资料，非经本人同意，不得外泄”。

句容市社区诊断居民健康调查表

（2015）

个人问卷

被调查个人姓名：________ 身份证号码：□□□□□□□□□□□□□□□□□□		
CODE1	个人编码	□□/□□/□□/□□
CODE2	调查结果	1　完成调查<br>2　部分完成调查<br>3　拒绝接受调查<br>88 其他，请说明________
调查员签名：________<br>日期：□□□□年□□月□□日		监测点质控员签名：________<br>日期：□□□□年□□月□□日
市级质控员签名：________<br>日期：□□□□年□□月□□日		
**调查员和质控员在完成个人问卷调查后填写以上 3 部分信息**		

句容市疾病预防控制中心

二〇一五年十月

Time1 调查开始时间□□时:□□分

第一部分　人口统计学

A1	您的出生日期	□□□□/□□/□□<br>年 / 月 / 日
A2	性别	1 男　2 女
A3	您的民族	1 汉族 88 其他，请说明____________
A4	您的文化程度	1 未接受正规学校教育<br>2 小学没毕业<br>3 小学毕业<br>4 初中毕业<br>5 高中/中专毕业<br>6 大专/本科毕业<br>7 研究生毕业及以上
A5	您目前的婚姻状况	1 单身 2 未婚同居 3 已婚分居 4 在婚 5 丧偶 6 离婚<br>结婚年龄为□□周岁（选 3-6 者填）
A6	您的职业	1 工人 2 农民 3 行政干部 4 科技、医务、教师<br>5 个体商企 6 家庭妇女 7 离退休人员 8 待业<br>9 学生　88 其他，请说明_____

第二部分　吸烟情况

现在吸烟情况			
B1	您现在是否吸烟（包括卷烟、手卷烟、烟斗、水烟、嚼烟、鼻烟等）？	1 是的，每天吸……………..……➔<br>2 是的，但不是每天吸………..…➔<br>3 不吸…………………………..……➔	B2<br>B3<br>B5
B2	您是从什么时候开始每天吸烟的？	□□周岁　-9 记不清	
B3	您是从什么时候开始吸第一支烟的？	□□周岁　-9 记不清	
B4	下列烟草，您通常一天吸多少？<br>调查员注意：记不清填“-9”，没有吸靠右填“0”。	1 香烟□□支<br>88 其他，请说明：______□□两/支<br>调查员注意：其他类型烟草包括手卷烟（支）、旱烟（两）、烟斗（两）、水烟（两）、雪茄（支）、非燃烧型烟草（如鼻烟、咀嚼烟等）（两）。	➔B7

戒烟行为			
B5	您过去是否吸烟？	1 是，每天吸<br>2 是，但不是每天吸<br>3 不吸 ……………………..………➔	B14
B6	您是什么时候停止吸烟的？	□□周岁 ………..…..………..….➔<br>-9 记不清 ………........………..…..…➔	B8<br>B8
B7	您目前是否打算戒烟？	1 是，下个月内<br>2 是，6 个月内<br>3 是，6 个月后<br>4 否，没打算戒烟<br>99 不知道	
B8	过去 12 个月里，您有没有为了尝试戒烟而停止使用烟草制品达 24 小时或更长时间？	1 有<br>2 没有 …………………………….➔<br>3 过去 12 个月未使用过烟草 …➔	B11<br>B13
B9	如果尝试过，有多少次？	□□次<br>-9 记不清	
B10	请回忆最近一次您为了尝试戒烟而停止使用烟草制品达 24 小时或更长时间的情况，停止了多长时间？<br>**调查员注意：只填 1 项**	□□月 或 □□周 或 □□天	

戒烟服务和戒烟产品			
B11	过去 12 个月里，在您看病或接受其他卫生保健服务的时候，有没有医生询问您有关吸烟的情况？	1 有<br>2 没有 …………………………….➔<br>3 没接受过医疗保健服务……..➔	B13<br>B13
B12	过去 12 个月里，在你看病时或接受其他卫生保健服务的时候，医生有没有建议您戒烟？	1 有<br>2 没有	
B13	您有没有用过以下方法来帮助您戒烟？<br>（可多选）	1 没有使用任何戒烟方法<br>2 戒烟门诊<br>3 尼古丁贴片、口含片、口香糖等<br>4 盐酸安非他酮类药物（如“悦亭”等）<br>5 其他处方药，请说明__________<br>6 中药<br>7 针灸<br>8 催眠<br>9 戒烟热线<br>10 电子雾化烟雾器<br>88 其他戒烟方法，请说明__________	

被动吸烟（二手烟暴露）			
B14	通常一周内，您在一天里吸入别人吸烟产生的烟雾（被动吸烟，吸二手烟）累计超过 15 分钟的情况有几天？	1 几乎没有 ………………………..➔<br>2 有，为 □ 天	B16

B15	您通常会在哪些地方被动吸烟？<br>**（可多选）**	1 家里<br>2 工作场所<br>3 餐厅<br>4 娱乐场所<br>5 公共交通工具及其等候室<br>88 其他，请说明____________

**对吸烟的知识和态度**				
B16	下面我要告诉您一些可能是或可能不是由吸烟或被动吸烟引起的健康问题和疾病。就您所知道的情况来看：			
		会	不会	不知道/不确定
吸烟会不会导致				
a	脑卒中	1	2	9
b	肺癌	1	2	9
c	心血管系统疾病（如高血压、冠心病）	1	2	9
d	白内障	1	2	9
e	流产	1	2	9
f	低出生体重儿	1	2	9
被动吸烟会不会导致				
g	肺癌	1	2	9
h	心血管系统疾病（如高血压、冠心病）	1	2	9

B17	对于以下说法，请告诉我您的态度：						
		非常同意	同意	无所谓	不同意	非常不同意	不知道/不确定
a	标明低焦油含量香烟的危害比一般香烟小	1	2	3	4	5	9
b	吸烟的人吸的每支烟都有害他们的健康	1	2	3	4	5	9
c	烟草会让人上瘾	1	2	3	4	5	9
d	公共场所应该禁止吸烟	1	2	3	4	5	9
e	政府应该加大控烟的力度	1	2	3	4	5	9

第三部分　饮酒与饮茶情况

饮酒			
C1	过去 12 个月里，您喝过酒吗？	1 喝过<br>2 没喝过 ……………………→	C9
C2	过去 12 个月里，您至少喝半两高度白酒，或 1 两低度白酒，或 1 易拉罐啤酒，或 1 两半黄酒，或 3 两葡萄酒的情况如何？	1 每天<br>2 5-6 天/周<br>3 3-4 天/周<br>4 1-2 天/周<br>5 1-3 天/月<br>6 少于 1 天/月	
C3	过去 12 个月里，在您喝酒的日子里，通常一天喝多少？<br>**调查员注意：记不清靠右填“-9”，没有喝靠右填“0”。**		
	a. 白酒（≥42 度）	□□□两	
	b. 白酒（<42 度）	□□□两	
	c. 啤酒	□□□瓶	
	d. 黄酒、糯米酒	□□□两	
	e. 葡萄酒	□□□两	
C4	过去 30 天里，您喝过酒吗？	1 喝过，为□天<br>2 没喝过……………………→	C7
C5	过去 7 天里，您喝过酒吗？	1 喝过，为□天<br>2 没喝过……………………→	C7
C6	过去 7 天里，以下所列酒类，您分别总共喝了多少？<br>**调查员注意：记不清右填“-9”，没有喝靠右填“0”。**		
	a. 白酒（≥42 度）	□□□两	
	b. 白酒（<42 度）	□□□两	
	c. 啤酒	□□□瓶	
	d. 黄酒、糯米酒	□□□两	
	e. 葡萄酒	□□□两	

C7	过去 12 个月里，以下各种类型的酒，您分别喝得最多的一次为多少？<br>**调查员注意：记不清右填“-9”，没有喝靠右填“0”。**				
			是否同时喝其他酒类<br>1=是 2=否	如果同时喝过其他酒<br>类型	饮酒量
	a. 白酒（≥42 度）	□□□两			□□□两/瓶
	b. 白酒（<42 度）	□□□两			□□□两/瓶
	c. 啤酒	□□□瓶			□□□两/瓶
	d. 黄酒、糯米酒	□□□两			□□□两/瓶
	e. 葡萄酒	□□□两			□□□两/瓶

C8	**a.对男性：**<br>过去 12 个月里，您有多少天一次喝酒超过5个标准饮酒单位？<br>调查员注意：标准饮酒单位换算见右侧酒精换算表。	□□□天<br>-9 记不清

附：酒精换算表 1

种　类	1个标准饮酒单位相当于	4个标准饮酒单位相当于	5个标准饮酒单位相当于
高度白酒(≥42度)	0.5 两	2 两	2 两半
低度白酒(<42度)	0.7 两	3 两	3 两半
啤酒（640ml 一瓶）	半瓶	2 瓶	2 瓶半
啤酒（355ml 一罐）	1 易拉罐	4 罐	5 罐
黄酒	1.5 两	6 两	7 两半
葡萄酒	3 两（150ml）	1 斤 2 两	1 斤半

C8	**b.对女性：**<br>过去 12 个月里，您有多少天一次喝酒超过4个标准饮酒单位？<br>调查员注意：标准饮酒单位换算见右侧酒精换算表。	□□□天<br>-9 记不清

附：酒精换算表 2

种　类	相当于标准饮酒单位（个）
**白酒：**	
1 两高度白酒（≥42 度）	2
1 斤高度白酒（≥42 度）	20
1 两低度白酒（<42 度）	1.5
1 斤低度白酒（<42 度）	15
**啤酒：**	
1 瓶啤酒（640ml）	2
1 易拉罐啤酒（355ml）	1
**黄酒（16 度）：**	
1 杯黄酒（80ml）	1
1 瓶（500ml）	6.5
**红酒：**	
1 红酒杯干红（120ml）	1
1 瓶干红（700ml）	6
1 瓶红葡萄酒（700ml）	4

饮茶

C9	您有饮茶的习惯吗？<br>调查员注意：此处的茶是指茶叶泡制的饮料，并非指白开水。	1　有（每周饮茶≥3 次）<br>2　偶尔(每周饮茶<3 次)<br>3　没有.................................➔	 D1<br>D1
C10	您多大年龄开始有饮茶习惯的？(指每天有意识泡茶)	□□岁	
C11	您最常喝哪种茶？	1 绿茶 2 红茶 3 花茶<br>88 其他，请说明__________	
C12	平均每天泡几次（杯）茶？	□□杯	
C13	平均每年（或月）消费几斤茶？<br>调查员注意：只填 1 项	□□斤/年 或 □□斤/月	

第四部分　饮食情况

D1	过去 12 个月里，您通常一周里吃早餐的天数？	1 每天都吃<br>2 5-6 天<br>3 3-4 天<br>4 1-2 天<br>5 不吃
D2	过去 12 个月里，通常您一天吃几餐？	□ 餐
D3	您通常一周内在外就餐的次数？（早餐除外）	□□ 次
D4	您通常一周内有几天吃水果？	□ 天
D5	您通常一周内吃多少种水果？	□ 种
D6	您通常一周内有几天吃蔬菜？	□ 天
D7	您通常一天内吃多少种蔬菜？	□ 种

请回忆在过去 12 个月里，您是否吃过下列食物，并估计各类食物的食用频率。

		a 是否食用<br>1 是，2 否	b 食用频率（**只填其中 1 项**）				
			b1 次数/天	b2 次数/周	b3 次数/月	b4 次数/年	平均每次食用量（两）
D8	谷类（米、面等）						□□.□ 两
D9	杂粮（小米、玉米、高粱等）						□□.□ 两
D10	薯类（红薯、山药、芋头、土豆等）						□□.□ 两
D11	蛋类						□□.□ 个
D12	鱼虾类						□□.□ 两
D13	畜肉类						□□.□ 两
D14	禽肉类						□□.□ 两

D15	奶类及其制品						□□.□ 两
D16	豆类及其制品						□□.□ 两

第五部分　身体活动

下面我要询问您进行各类身体活动的情况。请回答下列问题（即使您认为自己并不经常进行身体活动）。身体活动有很多种，包括干农活、工作、家务、交通相关的身体活动、休闲性锻炼或运动等。			
E1	在过去 12 个月里，您干过农活吗？	1 没有……………………………➔<br>2 干过，但没有农忙………➔<br>3 干过，有农忙、农闲之分	E4<br>E5
**农忙时的农业性身体活动**			
E2	在过去 12 个月里，您农忙时干农活的时间有几个月？<br>**调查员注意：不到 1 个月填“00”**	□□ 月	
E3	在农忙时干农活的日子里，您通常一天进行持续 10 分钟以上高强度活动的时间累计有多长?<br>（高强度活动是指如手工收割、挖掘、耕地等需要付出较大体力，或引起呼吸、心跳显著增加的活动）	□□小时 □□分钟….➔	E5
**工作、农业及家务性身体活动**<br>以下问题不涉及上述已提及的农忙时的身体活动情况，但包括农闲时以及没有农忙农闲之分的农业性身体活动。			
E4	您有没有从事其他工作、学习或者家务劳动?	1 有<br>2 没有………………………………➔	E11
E5	在您的工作、农活及家务活动中，有没有高强度活动，并且活动时间持续 10 分钟以上？<br>（高强度活动是指如搬运重物、挖掘等需要付出较大体力，或引起呼吸、心跳显著增加的活动）	1 有<br>2 没有 ……………………➔	E8
E6	在您的工作、农活及家务活动中，通常一周内有多少天会进行上述高强度活动?	□ 天	
E7	在您的工作、农活及家务活动中，通常一天内累计有多长时间进行上述高强度活动?<br>**调查员注意：每次活动时间若少于 10 分钟，则不计算在内。**	□□ 小时 □□ 分钟	
E8	在您的工作、农活及家务活动中，有没有中等强度活动，并且活动时间持续 10 分钟以上？<br>（中等强度活动是指如锯木、洗衣、打扫卫生等需要付出中等体力，或引起呼吸、心跳轻度增加的活动）	1 有<br>2 没有 ……………………➔	E11
E9	在您的工作、农活及家务活动中，通常一周内有多少天会进行上述中等强度活动?	□ 天	
E10	在您的工作、农活及家务活动中，通常一天内累计有多长时间进行上述中等强度活动?<br>**调查员注意：每次活动时间若少于 10 分钟，则不计算在内。**	□□ 小时 □□ 分钟	

**交通性身体活动**<br>以下问题不包括上述已提及的农业性身体活动和工作及家务性身体活动。对于干过农活，且有农忙农闲之分调查对象，本部分只涉及农闲时的交通性身体活动。			
E11	您去某个地方时，有没有步行或骑自行车持续至少 10 分钟的情况？	1 有<br>2 没有 ……………………→	E14
E12	通常一周内，您有多少天外出时步行或骑自行车持续至少 10 分钟？	□ 天	
E13	通常一天内，您步行或骑自行车多长时间？<br>**调查员注意：每次活动时间若少于 10 分钟，则不计算在内。**	□□ 小时 □□ 分钟	
**休闲性身体活动**<br>以下问题不包括上述已提及的农业性、工作、家务和交通性的身体活动。对于干过农活，且有农忙、农闲之分的调查对象，本部分只涉及农闲时的休闲性身体活动。			
E14	您是否进行持续至少 10 分钟，引起呼吸、心跳显著增加的剧烈运动或休闲活动吗？如长跑、踢足球等。	1 有<br>2 没有 ……………………→	E17
E15	通常一周内，您有多少天进行上述剧烈的运动或休闲活动？	□ 天	
E16	通常一天内，您累计有多长时间进行上述剧烈的运动或休闲活动？<br>**调查员注意：每次活动时间若少于 10 分钟，则不计算在内。**	□□ 小时 □□ 分钟	
E17	您是否进行持续至少 10 分钟，引起呼吸、心跳轻度增加的中等强度运动或休闲活动吗？如快步走、游泳、打排球等。	1 有<br>2 没有…………………...……→	F1
E18	通常一周内，您有多少天进行上述中等强度的运动或休闲活动？	□ 天	
E19	通常一天内，您累计有多长时间进行上述中等强度的运动或休闲活动？<br>**调查员注意：每次活动时间若少于 10 分钟，则不计算在内。**	□□ 小时 □□ 分钟	

附：常见不同强度身体活动分类表

农活、工作及家务中的身体活动		休闲性身体活动		静态行为
**中等强度身体活动**<br>使呼吸、心跳轻度加快	**剧烈身体活动**<br>使呼吸、心跳明显加快	**中等强度身体活动**<br>使呼吸、心跳轻度加快	**剧烈身体活动**<br>使呼吸、心跳明显加快	睡眠时间之外的坐着、靠着或躺着
**例：**<br>• 清洁（如吸尘、拖地、抛光地板、擦桌子、扫地、熨衣服）<br>• 洗涤（刷洗地毯、手洗衣物等）<br>• 园艺工作（如浇水、翻土、施肥等）<br>• 手工挤牛奶<br>• 农活（种植、收割庄稼等）<br>• 手工编织<br>• 木工（錾、锯软木材）	**例：**<br>• 林业工人（砍伐、搬运木材）<br>• 锯切硬木、耕地、插秧<br>• 收割庄稼（小麦、水稻、甘蔗等）<br>• 园艺工作（挖掘、搬重物等）<br>• 人工碾磨（用槌子或石磨等）<br>• 建筑工种工作（如搬运建筑材料、砌墙等）<br>• 搬运重物（如粮食、水	**例：**<br>• 骑车<br>• 慢跑<br>• 跳舞<br>• 骑马<br>• 打太极拳<br>• 练瑜珈、普拉提等柔缓健身运动<br>• 练低冲击健身操	**例：**<br>• 长跑<br>• 踢足球<br>• 打橄榄球<br>• 打网球<br>• 高冲击健身操<br>• 跳健美操、骑动感单车等剧烈绿色有氧运动<br>• 举哑铃、杠铃等肌肉锻炼运动<br>• 跳芭蕾舞<br>• 快速游泳	**例：**<br>• 工作<br>• 学习<br>• 阅读<br>• 看电视<br>• 用电脑<br>• 做手工活<br>• 乘坐机动车<br>• 坐着打牌、聊天等休息

• 用铣、铲等工具和水泥、沙子等<br>• 携带一般重量的东西行走<br>• 提/担水<br>• 放养家畜	泥或其他较重货物）<br>• 健身教练（如动感单车、健美操、瑜珈等有氧运动）<br>• 步行或骑车工作的速递工人<br>• 拉、蹬人力车、推独轮车、操作手提钻等			

第六部分　体重控制

F1	您最近一次测量体重的时间是？	1 从未量过……….➔<br>2 1 个月内<br>3 3 个月内<br>4 6 个月内<br>5 12 个月以内<br>6 12 个月以前<br>99 记不清	G1
F2	您的体重与 12 个月前相比有什么变化吗？	1 增加了 2.5 公斤或以上<br>2 基本保持不变（增减在 2.5 公斤以内）<br>3 下降了 2.5 公斤或以上<br>99 不知道	
F3	您认为自己现在的体重怎么样？	1 太瘦<br>2 有点瘦<br>3 标准水平<br>4 有点胖<br>5 太胖<br>99 没想过/不知道	
F4	过去 12 个月里，您是否采取过措施控制体重？	1 采取了措施来减轻体重<br>2 采取了措施来增加体重……….➔<br>3 未采取任何措施……………….➔	 G1<br>G1
F5	您减轻体重的方法有哪些？（可多选）	1 控制饮食总量<br>2 低脂饮食<br>3 低热量饮食<br>4 体育锻炼<br>5 药物<br>88 其他，请说明________________	

第七部分　健康状况

**高血压**			
G1	您最近一次测量血压距离现在有多长时间？	1 30 天内<br>2 1-6 个月<br>3 7-12 个月<br>4 12 个月以前<br>5 从来没测过血压……………➔<br>99 记不清	G5
G2	您有没有被乡镇/社区级或以上医院的医生诊断过患有高血压？	1 有<br>2 没有…………………………➔<br>99 不清楚	G5
G3	您首次被诊断高血压年龄或时间是？	□□周岁 或 □□□□年□□月	
G4a	您有没有采取措施来控制血压？	1 有<br>2 没有…………………………➔	G5
G4b	您采取了什么措施来控制血压?<br>**（可多选）**	1 按医嘱服药<br>2 控制饮食<br>3 适量运动<br>88 其他，请说明______________	
**糖尿病**			
G5	您最近一次测量血糖距离现在有多长时间？	1 30 天内<br>2 1-6 个月<br>3 7-12 个月<br>4 12 个月以前<br>5 从来没测过血糖……………➔<br>99 记不清	G9
G6a	您有没有被乡镇/社区级或以上医院的医生诊断患有糖尿病？<br>**调查员注意：不包括妊娠期糖尿病。**	1 有<br>2 没有…………………………➔<br>99 不清楚	G9
G6b	被诊断糖尿病类型	1 1 型<br>2 2 型…………………………➔<br>99 不清楚	G9
G7	您首次被诊断糖尿病年龄或时间是？	□□周岁 或 □□□□年□□月	

G8a	您有没有采取措施控制血糖？	1 有<br>2 没有……………………..………...➔	G9
G8b	您采取了什么措施来控制血糖?<br>（可多选）	1 按医嘱服药<br>2 控制饮食<br>3 适量运动<br>88 其他，请说明 ______________	

其它慢性病

G9	您或您的亲属有没有被诊断为以下疾病？<br>家族史亲属代码：1 父亲 2 母亲 3 爱人 4 哥哥 5 姐姐 6 弟弟 7 妹妹 8 姑姨 9 叔舅 10（外）祖父母		1 有<br>2 没有……………………...➔		G10
	疾病名称	您本人是否被诊断	具体类型	发病年龄或日期	家族史情况（可多选）
	a.高血压				
	b.糖尿病				
	c.血脂异常	1 是 2 否	1 高胆固醇血症（TC 升高）<br>2 高低密度脂蛋白血症（LDL 高）<br>3 低高密度脂蛋白血症（HDL 低）<br>4 高三酰甘油血症（TG 高）	□□周岁 或<br>□□□□年□□月	
	d.冠心病	1 是 2 否	1 心绞痛 2 心肌梗死<br>3 心律失常	□□周岁 或<br>□□□□年□□月	
	e.脑卒中	1 是 2 否	1 出血性脑卒中<br>2 缺血性脑卒中	□□周岁 或<br>□□□□年□□月	
	f.慢性阻塞性肺病（COPD）	1 是 2 否		□□周岁 或<br>□□□□年□□月	
	g.恶性肿瘤	1 是 2 否	肿瘤名称：______________<br>ICD 编码：C□□. □<br>病理类型：______________	□□周岁 或<br>□□□□年□□月	
	h.病毒性肝炎	1 是 2 否	1 甲肝 2 乙肝 3 丙肝<br>4 丁肝 5 戊肝	□□周岁 或<br>□□□□年□□月	
	i.脂肪性肝病	1 是 2 否	1 非酒精性<br>2 酒精性	□□周岁 或<br>□□□□年□□月	
	j.胃肠道疾病	1 是 2 否	疾病名称：______________	□□周岁 或<br>□□□□年□□月	
	k.肾脏病	1 是 2 否	疾病名称：______________	□□周岁 或<br>□□□□年□□月	
	l.风湿性关节炎	1 是 2 否		□□周岁 或<br>□□□□年□□月	
	m.其它呼吸系统疾病	1 是 2 否	疾病名称：______________	□□周岁 或<br>□□□□年□□月	
	n.其它血管病	1 是 2 否	疾病名称：______________	□□周岁 或<br>□□□□年□□月	
	o.其它疾病 1	1 是 2 否	疾病名称：______________	□□周岁 或<br>□□□□年□□月	
	p.其它疾病 2	1 是 2 否	疾病名称：______________	□□周岁 或<br>□□□□年□□月	
	q.其它疾病 3	1 是 2 否	疾病名称：______________	□□周岁 或<br>□□□□年□□月	

**女性宫颈癌和乳腺癌筛查（仅限女性）**<br>若为男性……………………………………………………………………………………➔	H1

G10	您有没有做过宫颈涂片检查？	1 有<br>2 没有……………………..….➔<br>99 不知道 ….……………..➔	 G12<br>G12
G11	如果做过，最近一次检查是在什么时候？<br>调查员注意：不到 1 年填“00”。	□□年前	
G12	您有没有做过乳腺 X 线检查？	1 有<br>2 没有 ⋯⋯⋯⋯⋯⋯⋯⋯➔<br>99 不知道 ……………..……➔	 G14<br>G14
G13	如果做过，最近一次检查是在什么时候？<br>调查员注意：不到 1 年填“00”。	□□年前	
G14	您有没有做过乳腺超声检查？	1 有<br>2 没有 ⋯⋯⋯⋯⋯⋯⋯⋯➔<br>99 不知道 ……………..……➔	 G16<br>G16
G15	如果做过，最近一次检查是在什么时候？<br>调查员注意：不到 1 年填“00”。	□□年前	
G16	您的月经初潮年龄为？	□□周岁	
G17	您是否已绝经？	1 没有<br>2 有，绝经年龄□□周岁<br>3 手术（于□□岁切除子宫，于□□岁切除卵巢）<br>99 不知道	

常用药情况

G18	您过去 1 年内是否服用过钙制剂？	1 是 2 否	
G19	您过去 1 年内是否服用过药物？	1 是<br>2 否 ⋯⋯⋯⋯⋯⋯⋯⋯➔	H1
G20	请回忆您过去 1 年内服用的药物（含钙制剂）情况。		

药物类型	药品名称		用药频率		用药剂量	药品生产厂商	主治或者预防疾病（自述）
	化学名	商品名	1. 每周<br>2. 每月<br>3. 每年	次数			
G20a 片剂 1					___次/日 ___片/次 ___mg/片		
G20b 片剂 2					___次/日 ___片/次 ___mg/片		
G20c 片剂 3					___次/日 ___片/次 ___mg/片		
G20d 片剂 4					___次/日 ___片/次 ___mg/片		
G20e					___次/日 ___片/次 ___mg/片		

片剂 5							
G20f<br>水剂					___次/日 ___ml/次		

第八部分　损伤与疼痛

过去 1 年内损伤情况							
H1	您过去 1 年内是否有损伤情况			1 有<br>2 没有 ⋯⋯⋯⋯⋯⋯→		H2	
损伤类型	部位<br>1 头颈部<br>2 面部<br>3 上肢<br>4 下肢<br>5 躯干<br>6 手指/脚趾		外部原因<br>1 运输事故<br>2 跌倒/坠落<br>3 碰撞/挤压<br>4 扭伤<br>5 割伤/刺伤<br>6 动物咬伤<br>7 淹溺/沉没<br>8 窒息<br>9 电击伤<br>10 热/火烫<br>11 爆炸<br>12 其他, 请说明<br>99 不详	内部原因<br>1 浅表伤<br>2 开放性伤<br>3 脱位/扭伤/劳损<br>4 挤压伤<br>5 神经/脊髓伤<br>6 器官系统伤<br>7 多种性质________<br>8 其他，请说明___<br>99 不知道	伤害时间	地点<br>1 家中/宿舍<br>2 公共场所<br>3 工作场所<br>99 不知道	对功能影响<br>1 无影响<br>2 轻微<br>3 影响部分功能<br>4 严重影响<br>5 不能自理
	左侧	右侧					
H1a.骨折 1				——	___年__月		
H1b.骨折 2				——	___年__月		
H1c.骨折 3				——	___年__月		
H1d.摔伤 1					___年__月		
H1e.摔伤 2					___年__月		
H1f.摔伤 3					___年__月		
H1g.其它 1					___年__月		
H1h.其它 2					___年__月		
H1i.其它 3					___年__月		
**过去 1 年内疼痛情况**							
H2	您过去 1 年内是否有疼痛情况			1 有<br>2 没有 ⋯⋯⋯⋯⋯⋯→		I1	
**疼痛类型**	**部位**<br>1 头颈部<br>2 面部<br>3 上肢<br>4 下肢<br>5 躯干<br>6 手指/脚趾		**疼痛原因**<br>1 外伤<br>2 疾病<br>3 情感刺激<br>4 其它，请说明<br>______	**疼痛频次**<br>1 七天内<br>2 30 天内<br>3 1-6 个月<br>4 7-12 个月	**疼痛程度**<br>1 无<br>2 轻<br>3 中<br>4 重	**处理措施**<br>1 未处理<br>2 内服药<br>3 外敷药<br>4 理疗	**对功能影响**<br>1 无影响<br>2 轻微<br>3 影响部分功能<br>4 严重影响<br>5 不能自理
	左侧	右侧					
H2a.疼痛 1							

H2b.疼痛 2							
H2c.疼痛 3							

第九部分　睡眠状况

**睡眠时间与质量**		
下面问题有助于对您的睡眠习惯和睡眠质量进行评估		
I1	近 1 个月，晚上上床睡觉时间通常是几点钟？	□□时 □□分
I2	近 1 个月，从上床到入睡通常需要多少分钟？	□□□分钟
I3	近 1 个月，通常早上几点起床？	□□时 □□分
I4a	近 1 个月，每夜通常实际睡眠时间几小时？	□□小时 □□分钟
I4b	近 1 个月，中午及其他时间睡眠	□□小时 □□分钟
对下列问题选择请选择一个最合适您的答案。		
近一个月，您有没有因下列情况影响睡眠而烦恼？		
I5a	入睡困难（30 分钟内不能入睡）	1 无　2 <1 次/周　3 1~2 次/周　4≥3 次/周
I5b	夜间易醒或早醒	1 无　2 <1 次/周　3 1~2 次/周　4≥3 次/周
I5c	夜间去厕所	1 无　2 <1 次/周　3 1~2 次/周　4≥3 次/周
I5d	呼吸不畅	1 无　2 <1 次/周　3 1~2 次/周　4≥3 次/周
I5e	咳嗽或鼾声高（因打鼾影响呼吸）	1 无　2 <1 次/周　3 1~2 次/周　4≥3 次/周
I5f	感觉冷	1 无　2 <1 次/周　3 1~2 次/周　4≥3 次/周
I5g	感觉热	1 无　2 <1 次/周　3 1~2 次/周　4≥3 次/周
I5h	做噩梦	1 无　2 <1 次/周　3 1~2 次/周　4≥3 次/周
I5i	疼痛不适	1 无　2 <1 次/周　3 1~2 次/周　4≥3 次/周
I5j	其他影响睡眠的事情	1 无　2 <1 次/周　3 1~2 次/周　4≥3 次/周<br>如果有，请说明＿＿＿＿＿＿＿＿＿
I6	近 1 个月，总的来说，您认为自己的睡眠质量	1 很好　2 较好　3 较差　4 很差
I7	近 1 个月，您用催眠药物的情况	1 无　2 <1 次周　3 1~2 次周　4≧3 次周
I8	近 1 个月，您感到困倦吗?	1 无　2 <1 次周　3 1~2 次周　4≧3 次周
I9	近 1 个月，您感到做事的精力不足吗	1 没有 2 偶尔有　3 有时有 4 经常有

**不宁腿症状**			
您是否有以下症状？			
I10	您的腿是否会有像蚂蚁爬行、电击、刺痛、灼烧的感觉，这些感觉让您非常想要活动活动腿？	1 是 □<br>2 否……………………………→	J1
I11	当您静坐或躺着时这些感觉是否加重？	1 是 □<br>2 否……………………………→	J1
I12	当您走动的时候这些感觉是否减轻？	1 是 □<br>2 否……………………………→	J1
I13	您的这些感觉晚上是否比白天更严重？	1 是 □<br>2 否……………………………→	J1
I14	您的这些感觉是否每次都是由于腿抽筋引起的？□	1 是……………………………→<br>2 否	J1
I15	您的这些感觉是否换换腿的姿势后就会减轻？	1 通常会减轻……………→<br>2 偶尔会减轻 □<br>3 不会减轻	J1
I16	您经常会有这些感觉吗？	1 一个月 1 次或少于 1 次 □2 一个月 2 到 4 次 □<br>3 一个星期 2 到 3 次　4 一个星期 4 到 5 次<br>5 一个星期 6 到 7 次 □　6 更多	
I17	上述症状您持续多长时间？	□□年	

Time2 调查结束时间 □□时:□□分

身体测量记录表

个人编码：□□/□□/□□/□□

**身高、体重和腰围知晓情况**		
**您好，下面我们会问您几个关于身高和体重的问题。**		
J1	您知道自己的身高吗？	1 知道，□□□.□ 厘米(cm) 2 不知道
J2	您知道自己的体重吗？	1 知道，□□□.□ 千克(kg) 2 不知道
J3	您知道自己的腰围吗？	1 知道，□□□.□ 厘米(cm) 2 不知道
J4	您知道自己的臀围吗？	1 知道，□□□.□ 厘米(cm) 2 不知道
**身体指标测量**		
**您好，下面我们将测量您的身高、体重、腰围、臀围和颈围，请您配合。**		
K1	身高	□□□.□ 厘米(cm)
K2	体重	□□□.□ 公斤(kg)
K3	K3a 上颈围	□□.□ 厘米(cm)
	K3b 下颈围	□□.□ 厘米(cm)
K4	腰围	□□□.□ 厘米(cm)
K5	臀围	□□□.□ 厘米(cm)

**您好，下面我们将测量您的血压和心率，请您配合。**			
Time3	测量开始时间：□□时 □□分		
K6	室内温度：□□. □℃	K7	血压计编号：□□
K8a	第 1 次读数<br>**调查员注意：测量对象休息 15 分钟后第 1 次测量并记录血压，休息 30 秒后第 2 次测量血压和心率**	收缩压	□□□（mmHg）
K8b		舒张压	□□□（mmHg）
K8c		心率	□□□ 次/分
K9a	第 2 次读数	收缩压	□□□（mmHg）

K9b	调查员注意：记录第 2 次测量结果，待测量对象再休息 30 秒后第 3 次测量血压和心率	舒张压	□□□（mmHg）□□
K9c		心率	□□□ 次/分
K10a	第 3 次读数<br>记录第 3 次测量结果	收缩压	□□□（mmHg）
K10b		舒张压	□□□（mmHg）
K10c		心率	□□□ 次/分
如果以上 3 次收缩压或舒张压测量值误差≥8mmHg，再进行第四次测量，并记录第四次血压和心率。			
K11a	第 4 次读数<br>记录第 4 次测量结果	收缩压	□□□（mmHg）
K11b		舒张压	□□□（mmHg）
K11c		心率	□□□ 次/分
Time4	测量结束时间: □□时□□分		

体能、体成分与骨密度测量

您好，下面我们将测量您的体能、体成分与骨密度，请您配合。

L1	体成分		结果内容较多，且会出具专门的报告单，此处不需填写。	
L2	**60 周岁以上居民参加**	握力	1 左侧 □□.□Kg	2 右侧 □□.□Kg
L3		拉力（伸膝）	1 左侧 □□.□Kg	2 右侧 □□.□Kg
		拉力（屈膝）	1 左侧 □□.□Kg	2 右侧 □□.□Kg
L4		平衡	结果内容较多，且会出具专门的报告单，此处不需填写。	
L5		6m 步速	□□.□ s	
L6		30s 连续坐起	□□ 次	
L7		3m 折返起立-走	□□.□ s	
L8		反应时（优势侧）	1 左侧　2 右侧	□□□□. □ms
L9	骨密度		1 骨量正常　2 低骨量　3 骨质疏松	
			T 值	+/− □.□□
			Z 值	+/− □.□□

2015 年句容市社区诊断居民健康调查
现场工作流转单

编号：□□/□□/□□/□□

姓名：__________性别：_____出生日期：____年___月___日（___周岁）

地址：______镇_____行政村（居委会）_____村　手机号：___________

序号	流程		是否完成（完成打“√”）	工作人员签名
1	登　记　编　号			
2	采　血			
3	人体测量	身高、体重		
		颈围、腰围、臀围		
4	血　压　测　量			
5	体能与体成分	体成分		
		握力		
		拉力		
		平衡		
		6m 步速		
		30s 连续坐起		
		3m 折返起立-走		
		反应时（优势侧）		
6	骨　密　度			
7	问　卷　调　查			
8	资　料　回　收			

调查日期：2015 年___月___日
