# Supplementary material for: Sex- and Age-Specific Prevalence of Osteopenia and Osteoporosis: Sampling Survey
Source: JMIR Public Health Surveill. 2024 Apr 5;10:e48947. doi: 10.2196/48947 (PMC11031699; doi:10.2196/48947)
Supplement: Multimedia Appendix 2 [file publichealth_v10i1e48947_app2.docx]

| **Multimedia Appendix 2.** Prevalence rates of osteopenia and osteoporosis among age-groups. | | | | | | | | | |
| --- | --- | --- | --- | --- | --- | --- | --- | --- | --- |
| Age group  (years) | Osteopenia | | | |  | Osteoporosis | | | |
|  | Total population | Male participants | Female participants | *P* value |  | Total population | Male participants | Female participants | *P* value |
|  | n (%) | n (%) | n (%) |  |  | n (%) | n (%) | n (%) |  |
| 18 | 120 (21.47) | 41 (20.71) | 79 (21.88) | .68 |  | 5 (0.89) | 0 (0) | 5 (1.39) | .09 |
| 40 | 159 (23.8) | 57 (23.17) | 102 (24.17) | .73 |  | 6 (0.9) | 1 (0.41) | 5 (1.18) | .3 |
| 45 | 467 (29.26) | 157 (28.44) | 310 (29.69) | .39 |  | 37 (2.32) | 3 (0.54) | 34 (3.26) | <.001 |
| 50 | 841 (33.8) | 297 (33.75) | 544 (33.83) | .37 |  | 110 (4.42) | 12 (1.36) | 98 (6.09) | <.001 |
| 55 | 732 (37.39) | 225 (29.45) | 507 (42.46) | <.001 |  | 145 (7.41) | 16 (2.09) | 129 (10.80) | <.001 |
| 60 | 1261 (41.67) | 428 (32.30) | 833 (48.97) | <.001 |  | 200 (6.61) | 27 (2.04) | 173 (10.17) | <.001 |
| 65 | 1351 (46.63) | 480 (38.1) | 871 (53.21) | <.001 |  | 291 (10.04) | 28 (2.22) | 263 (16.07) | <.001 |
| 70 | 948 (51.41) | 359 (43.46) | 589 (57.86) | <.001 |  | 274 (14.86) | 45 (5.45) | 229 (22.5) | <.001 |
| 75 | 754 (56.23) | 351 (51.54) | 403 (61.06) | <.001 |  | 231 (17.23) | 37 (5.43) | 194 (29.39) | <.001 |
| Total | 6633 (40.5) | 2395 (35.58) | 4238 (43.94) | <.001 |  | 1299 (7.93) | 169 (2.51) | 1130 (11.72) | <.001 |
