# Supplementary material for: Sex- and Age-Specific Prevalence of Osteopenia and Osteoporosis: Sampling Survey
Source: JMIR Public Health Surveill. 2024 Apr 5;10:e48947. doi: 10.2196/48947 (PMC11031699; doi:10.2196/48947)
Supplement: Multimedia Appendix 3 [file publichealth_v10i1e48947_app3.docx]

| **Multimedia Appendix 3.** Prevalence rates of osteopenia and osteoporosis in postmenopausal and premenopausal female participants. | | | | | | | |
| --- | --- | --- | --- | --- | --- | --- | --- |
| Age group  (years) | Osteopenia | | |  | Osteoporosis | | |
|  | Premenopausal | Postmenopausal | *P* value |  | Premenopausal | Postmenopausal | *P* value |
|  | n (%) | n (%) |  |  | n (%) | n (%) |  |
| 18 | 76 (21.41) | N/A^a^ |  |  | 5 (1.41) | N/A |  |
| 40 | 96 (24) | 5 (23.81) | .96 |  | 5 (1.25) | N/A |  |
| 45 | 223 (28.66) | 84 (32.43) | .11 |  | 16 (2.06) | 17 (6.56) | <.001 |
| 50 | 123 (27.64) | 416 (36.21) | <.001 |  | 21(4.72) | 76 (6.61) | .046 |
| 55 | 11 (32.35) | 493 (42.68) | .15 |  | 4 (11.76) | 125 (10.82) | .86 |
| 60 | 9 (64.29) | 816 (48.80) | .13 |  | 2 (14.29) | 168 (10.05) | .25 |
| 65 | N/A | 850 (53.26) |  |  | N/A | 256 (16.04) |  |
| 70 | N/A | 579 (58.02) |  |  | N/A | 223 (22.34) |  |
| 75 | N/A | 394 (61.37) |  |  | N/A | 188 (29.28) |  |
| Total | 538 (26.55) | 3638 (48.55) | <.001 |  | 53 (2.62) | 1053 (14.05) | <.001 |
|  |  |  |  |  |  |  |  |

^a^N/A: not applicable.
