# Supplementary material for: Sex- and Age-Specific Prevalence of Osteopenia and Osteoporosis: Sampling Survey
Source: JMIR Public Health Surveill. 2024 Apr 5;10:e48947. doi: 10.2196/48947 (PMC11031699; doi:10.2196/48947)
Supplement: Multimedia Appendix 4 [file publichealth_v10i1e48947_app4.docx]

| **Multimedia Appendix 4.** Prevalence rates of osteopenia and osteoporosis in subgroups with and without taking calcium. | | | | | | | | |
| --- | --- | --- | --- | --- | --- | --- | --- | --- |
| Population | Age group  (years) | Osteopenia | | |  | Osteoporosis | | |
|  |  | Taking calcium | Without taking calcium | *P* value |  | Taking calcium | Without taking calcium | *P* value |
|  |  | n (%) | n (%) |  |  | n (%) | n (%) |  |
| Total population | 18 | 5 (15.63) | 115 (21.82) | .39 |  | N/A | 5 (0.95) |  |
|  | 40 | 5 (35.71) | 154 (23.55) | .30 |  | N/A | 6 (0.92) |  |
|  | 45 | 14 (28.57) | 453 (29.28) | .90 |  | 1 (2.04) | 36 (2.33) | .89 |
|  | 50 | 50 (36.23) | 790 (33.65) | .58 |  | 5 (3.62) | 105 (4.47) | .7 |
|  | 55 | 46 (43.81) | 683 (36.96) | .23 |  | 5 (4.76) | 140 (7.58) | .41 |
|  | 60 | 77 (46.39) | 1183 (41.42) | .25 |  | 9 (5.42) | 191 (6.69) | .71 |
|  | 65 | 68 (49.28) | 1278 (46.51) | .63 |  | 12 (8.70) | 278 (10.12) | .71 |
|  | 70 | 36 (53.73) | 910 (51.33) | .67 |  | 10 (14.93) | 264 (14.89) | .84 |
|  | 75 | 40 (70.18) | 710 (55.47) | .10 |  | 6 (10.53) | 225 (17.58) | .73 |
|  | Total | 341 (44.52) | 6276 (40.28) | .05 |  | 48 (6.27) | 1250 (8.02) | .21 |
| Male participants | 18 | 1 (20) | 40 (20.73) | .97 |  | N/A | N/A |  |
|  | 40 | N/A^a^ | 57 (23.36) |  |  | N/A | 1 (0.41) |  |
|  | 45 | 5 (38.46) | 152 (28.2) | .43 |  | N/A | 3 (0.56) |  |
|  | 50 | 4 (22.22) | 293 (33.99) | .28 |  | N/A | 12 (1.39) |  |
|  | 55 | 5 (20.83) | 220 (29.77) | .37 |  | 1 (4.17) | 15 (2.03) | .55 |
|  | 60 | 11 (20.37) | 417 (32.83) | .06 |  | 2 (3.70) | 25 (1.97) | .52 |
|  | 65 | 15 (35.71) | 464 (38.25) | .65 |  | N/A | 28 (2.31) |  |
|  | 70 | 5 (23.81) | 354 (44.03) | .08 |  | 2 (9.52) | 43 (5.35) | .7 |
|  | 75 | 9 (50) | 342 (51.58) | .67 |  | 3 (16.67) | 34 (5.13) | .03 |
|  | Total | 55 (27.92) | 2339 (35.84) | .03 |  | 8 (4.06) | 161 (2.47) | .28 |
| Female participants | 18 | 4 (14.81) | 75 (22.46) | .34 |  | N/A | 5 (1.5) |  |
|  | 40 | 5 (41.67) | 97 (23.66) | .16 |  | N/A | 5 (1.22) |  |
|  | 45 | 9 (25) | 301 (29.86) | .52 |  | 1 (2.78) | 33 (3.27) | .82 |
|  | 50 | 46 (38.33) | 497 (33.45) | .35 |  | 5 (4.17) | 93 (6.26) | .45 |
|  | 55 | 41 (50.62) | 463 (41.75) | .30 |  | 4 (4.94) | 125 (11.27) | .14 |
|  | 60 | 66 (58.93) | 766 (48.3) | .08 |  | 7 (6.25) | 166 (10.47) | .41 |
|  | 65 | 53 (55.21) | 814 (53.03) | .96 |  | 12 (12.5) | 250 (16.29) | .36 |
|  | 70 | 31 (67.39) | 556 (57.38) | .32 |  | 8 (17.39) | 221 (22.81) | .99 |
|  | 75 | 31 (79.49) | 368 (59.64) | .96 |  | 3 (7.69) | 191 (30.96) | .01 |
|  | Total | 286 (50.26) | 3937 (43.48) | .04 |  | 40 (7.03) | 1089 (12.03) | .004 |

^a^N/A: not applicable.
