# Supplementary material for: Sex- and Age-Specific Prevalence of Osteopenia and Osteoporosis: Sampling Survey
Source: JMIR Public Health Surveill. 2024 Apr 5;10:e48947. doi: 10.2196/48947 (PMC11031699; doi:10.2196/48947)
Supplement: Multimedia Appendix 5 [file publichealth_v10i1e48947_app5.docx]

| **Multimedia Appendix 5.** Association analysis of history of taking calcium with osteopenia and osteoporosis. | | | | | |
| --- | --- | --- | --- | --- | --- |
| Group | Population | Osteopenia or Osteoporosis (n) | Normal BMD (n) | OR (95% CI) | OR (95% CI) |
| Osteopenia | Whole population | 6497 | 7998 | 1.03 (0.88-1.20)^a^ | 1.10 (0.94-1.29)^c^ |
|  |  |  |  | *P*=.76 | *P*=.25 |
|  | Male participants | 2394 | 4161 | 0.68 (0.50-0.94)^b^ | 0.75 (0.53-1.04)^d^ |
|  |  |  |  | *P*=.02 | *P*=.08 |
|  | Female participants | 4223 | 4271 | 1.18 (0.98-1.43)^b^ | 1.26 (1.04-1.52)^d^ |
|  |  |  |  | *P*=.08 | *P*=.02 |
| Osteoporosis | Whole population | 1298 | 8432 | 0.71 (0.51-0.99)^a^ | 0.90 (0.61-1.34)^c^ |
|  |  |  |  | *P*=.046 | *P*=.61 |
|  | Male participants | 169 | 4161 | 1.43 (0.68-3.00)^b^ | 1.82 (0.81-4.09)^d^ |
|  |  |  |  | *P*=.34 | *P*=.15 |
|  | Female participants | 1129 | 4271 | 0.63 (0.43-0.91)^b^ | 0.75 (0.48-1.16)^d^ |
|  |  |  |  | *P*=.01 | *P*=.19 |

^a^Adjusted for age and sex.

^b^Adjusted for age.

^c^Adjusted for age, sex, PAI, occupation, history of HTN, T2DM, dyslipidemia, CHD, stroke and cancer.

^d^Adjusted for age, PAI, occupation, history of HTN, T2DM, dyslipidemia, CHD, stroke and cancer.
