# Supplementary material for: Sex- and Age-Specific Prevalence of Osteopenia and Osteoporosis: Sampling Survey
Source: JMIR Public Health Surveill. 2024 Apr 5;10:e48947. doi: 10.2196/48947 (PMC11031699; doi:10.2196/48947)
Supplement: Multimedia Appendix 6 [file publichealth_v10i1e48947_app6.docx]

| **Multimedia Appendix 6.** Prevalence of chronic disease among different T-score levels. | | | | | | | | | |
| --- | --- | --- | --- | --- | --- | --- | --- | --- | --- |
|  | Variables | Level | The T value was grouped by four quantiles | | | | | |  |
|  |  |  | Overall | 1 | 2 | 3 | 4 | *P* value | *P _trend_* value |
| Total population | n |  | 15839 | 4037 | 4029 | 3894 | 3879 |  |  |
|  | Age (years), median (IQR) |  | 61.17 (52.42-68.00) | 61.75 (53.00-68.33) | 61.08 (52.42-68.00) | 61.25 (52.27-68.00) | 61.00 (52.08-67.75) | .25 | .07 |
|  | Sex, n (%) | Male participants | 6505 (41.07) | 1651 (40.9) | 1670 (41.45) | 1587 (40.76) | 1597 (41.17) | .93 | .97 |
|  |  | Female participants | 9334 (58.93) | 2386 (59.1) | 2359 (58.55) | 2307 (59.24) | 2282 (58.83) |  |  |
|  | HTN^a^ baseline, n (%) |  | 10121 (63.9) | 2630 (65.15) | 2511 (62.32) | 2503 (64.28) | 2477 (63.86) | .06 | .57 |
|  | DM^b^ baseline, n (%) |  | 3169 (20.01) | 752 (18.63) | 779 (19.33) | 769 (19.75) | 869 (22.4) | <.001 | <0.001 |
|  | Dyslipidemia, n (%) |  | 9217 (58.22) | 2228 (55.2) | 2304 (57.21) | 2306 (59.26) | 2379 (61.35) | <.001 | <0.001 |
|  | CHD^c^ baseline, n (%) | | 275 (1.74) | 52 (1.29) | 62 (1.54) | 84 (2.16) | 77 (1.99) | .01 | .003 |
|  | Stroke baseline, n (%) | | 855 (5.4) | 218 (5.40) | 200 (4.96) | 199 (5.11) | 238 (6.14) | .1 | .15 |
|  | Cancer baseline, n (%) | | 402 (2.54) | 112 (2.77) | 110 (2.73) | 103 (2.65) | 77 (1.99) | .09 | .03 |
|  | CCVD^d^ baseline, n (%) | | 1085 (6.85) | 261 (6.47) | 254 (6.3) | 269 (6.91) | 301 (7.76) | .05 | .01 |
| 18.5~24 | n |  | 6315 | 1786 | 1642 | 1552 | 1335 |  |  |
| (kg/m^2^) | Age, (years), median (IQR) |  | 61.75 (52.58-69.00) | 62.00 (53.00-69.08) | 61.46 (52.58-68.65) | 61.62 (52.50-68.58) | 61.83 (52.17-68.58) | .70 |  |
|  | Sex, n (%) | Male participants | 2719 (43.06) | 746 (41.77) | 698 (42.51) | 686 (44.2) | 589 (44.12) | .42 |  |
|  |  | Female participants | 3596 (56.94) | 1040 (58.23) | 944 (57.49) | 866 (55.8) | 746 (55.88) |  |  |
|  | HTN baseline, n (%) |  | 3399 (53.82) | 993 (55.6) | 859 (52.31) | 830 (53.48) | 717 (53.71) | .28 | 0.57 |
|  | DM baseline, n (%) |  | 878 (13.9) | 241 (13.49) | 214 (13.03) | 206 (13.27) | 217 (16.25) | .047 | <.001 |
|  | Dyslipidemia, n (%) |  | 3087 (48.91) | 865 (48.46) | 780 (47.53) | 763 (49.19) | 679 (50.86) | .32 | <.001 |
|  | CHD baseline, n (%) | | 83 (1.31) | 18 (1.01) | 23 (1.4) | 23 (1.48) | 19 (1.42) | .61 | .003 |
|  | Stroke baseline, n (%) | | 281 (4.45) | 77 (4.31) | 69 (4.2) | 64 (4.12) | 71 (5.32) | .38 | .15 |
|  | Cancer baseline, n (%) | | 219 (3.47) | 64 (3.58) | 66 (4.02) | 54 (3.48) | 35 (2.62) | .22 | .03 |
|  | CCVD baseline, n (%) | | 352 (5.57) | 91 (5.1) | 89 (5.42) | 85 (5.48) | 87 (6.52) | .37 | .01 |
| 24~28 | n |  | 6673 | 1641 | 1670 | 1628 | 1734 |  |  |
| (kg/m^2^) | Age, (years), median (IQR) |  | 61.00 (52.42-67.75) | 61.00 (53.00-68.00) | 61.12 (52.58-68.00) | 61.17 (52.23-67.67) | 60.79 (52.08-67.42) | .74 |  |
|  | Sex, n (%) | Male participants | 2765 (41.44) | 670 (40.83) | 704 (42.16) | 654 (40.17) | 737 (42.5) | .48 |  |
|  |  | Female participants | 3908 (58.56) | 971 (59.17) | 966 (57.84) | 974 (59.83) | 997 (57.5) |  |  |
|  | HTN baseline, n (%) |  | 4488 (67.26) | 1143 (69.65) | 1102 (65.99) | 1102 (67.69) | 1141 (65.80) | .06 | .57 |
|  | DM baseline, n (%) |  | 1436 (21.52) | 347 (21.15) | 355 (21.26) | 349 (21.44) | 385 (22.2) | .88 | <.001 |
|  | Dyslipidemia, n (%) |  | 4125 (61.84) | 968 (58.99) | 1014 (60.72) | 1023 (62.92) | 1120 (64.63) | .005 | <.001 |
|  | CHD baseline, n (%) | | 125 (1.87) | 29 (1.77) | 24 (1.44) | 42 (2.58) | 30 (1.73) | .09 | .003 |
|  | Stroke baseline, n (%) | | 379 (5.68) | 93 (5.67) | 88 (5.27) | 92 (5.65) | 106 (6.11) | .77 | .15 |
|  | Cancer baseline, n (%) | | 139 (2.08) | 37 (2.25) | 33 (1.98) | 34 (2.09) | 35 (2.02) | .95 | .03 |
|  | CCVD baseline, n (%) | | 486 (7.28) | 119 (7.25) | 111 (6.65) | 126 (7.74) | 130 (7.5) | .65 | .01 |
| BMI>28 | n |  | 2850 | 610 | 716 | 714 | 810 |  |  |
| (kg/m^2^) | Age, (years), median (IQR) |  | 61.00 (52.00-67.08) | 61.16 (53.00-67.00) | 60.83 (52.00-67.25) | 61.00 (52.00-67.17) | 60.50 (51.94-67.08) | .87 |  |
|  | Sex, n (%) | Male participants | 1021 (35.82) | 235 (38.52) | 268 (37.43) | 247 (34.59) | 271 (33.46) | .16 |  |
|  |  | Female participants | 1829 (64.18) | 375 (61.48) | 448 (62.57) | 467 (65.41) | 539 (66.54) |  |  |
|  | HTN baseline, n (%) |  | 2233 (78.35) | 494 (80.98) | 549 (76.68) | 571 (79.97) | 619 (76.42) | .09 | .57 |
|  | DM baseline, n (%) |  | 854 (29.96) | 164 (26.89) | 209 (29.19) | 214 (29.97) | 267 (32.96) | .09 | <.001 |
|  | Dyslipidemia, n (%) |  | 2005 (70.38) | 395 (64.75) | 510 (71.33) | 520 (72.83) | 580 (71.6) | .007 | <.001 |
|  | CHD baseline, n (%) | | 67 (2.35) | 5 (0.82) | 15 (2.09) | 19 (2.66) | 28 (3.46) | .01 | .003 |
|  | Stroke baseline, n (%) | | 195 (6.84) | 48 (7.87) | 43 (6.01) | 43 (6.02) | 61 (7.53) | .37 | .15 |
|  | Cancer baseline, n (%) | | 44 (1.54) | 11 (1.8) | 11 (1.54) | 15 (2.1) | 7 (0.86) | .24 | .03 |
|  | CCVD baseline, n (%) | | 247 (8.67) | 51 (8.36) | 54 (7.54) | 58 (8.12) | 84 (10.37) | .22 | .01 |

^a^HTN: hypertension.

^b^DM: diabetes mellitus.

^c^CHD: coronary heart disease.

^d^CCVD: cardiovascular and cerebrovascular disease.
