# Supplementary figures and images for: Sex- and Age-Specific Prevalence of Osteopenia and Osteoporosis: Sampling Survey
Source: JMIR Public Health Surveill. 2024 Apr 5;10:e48947. doi: 10.2196/48947 (PMC11031699; doi:10.2196/48947)

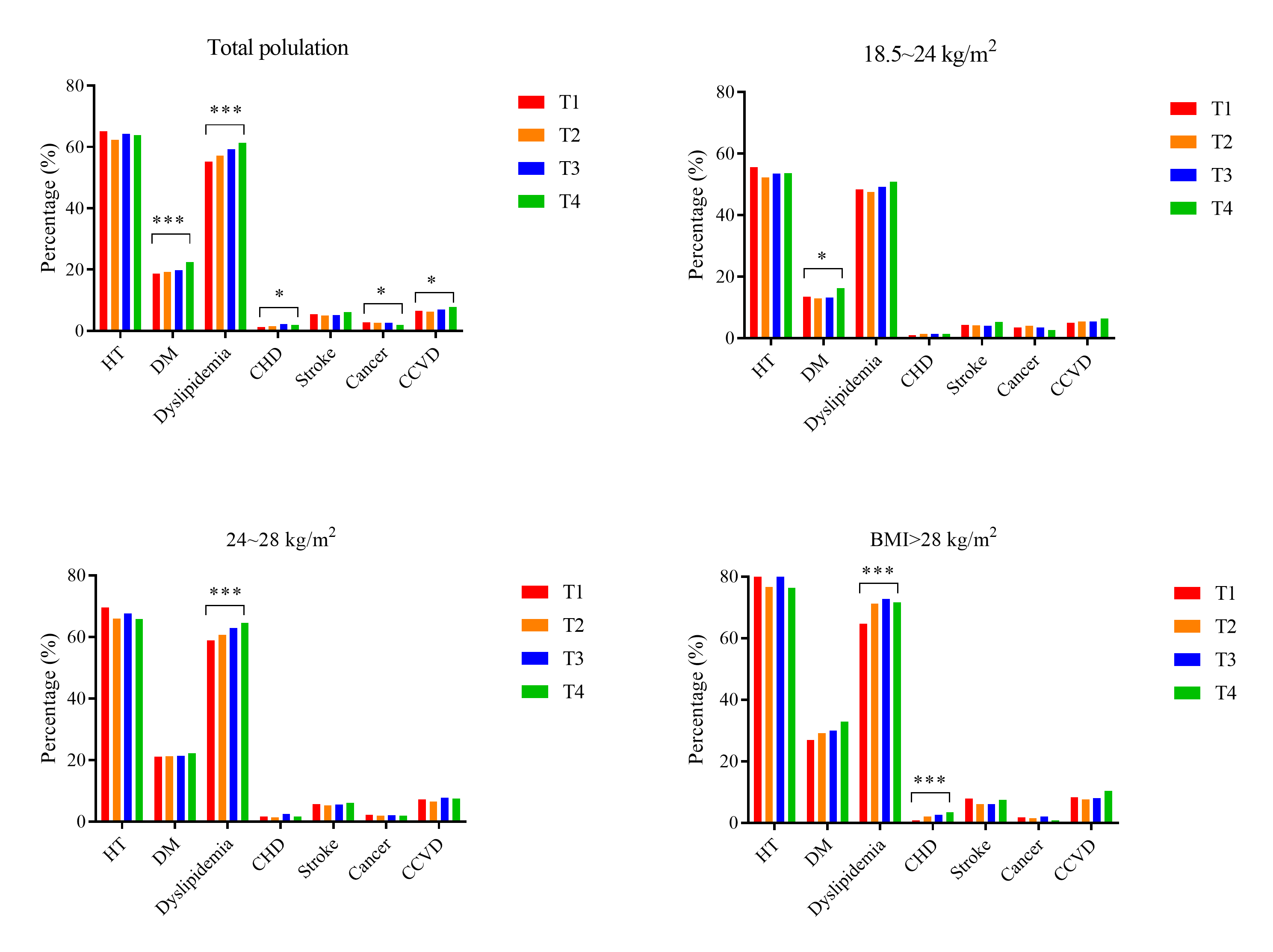

Supplement: Multimedia Appendix 7 [file publichealth_v10i1e48947_app7.png]
